# Identification of Lysine Histidine Transporter 2 as an 1-Aminocyclopropane Carboxylic Acid Transporter in *Arabidopsis thaliana* by Transgenic Complementation Approach

**DOI:** 10.3389/fpls.2019.01092

**Published:** 2019-09-11

**Authors:** Jungki Choi, Sanung Eom, Kihye Shin, Rin-A Lee, Soobin Choi, Jun-Ho Lee, Sumin Lee, Moon-Soo Soh

**Affiliations:** ^1^Division of Integrative Bioscience and Biotechnology, College of Life Science, Sejong University, Seoul, South Korea; ^2^Departments of Biotechnology, Chonnam National University, Gwangju, South Korea

**Keywords:** 1-aminocyclopropane carboxylic acid, amino acid transporter, triple response, LHT1, LHT2, transgenic complementation, *Arabidopsis thaliana*

## Abstract

1-Aminocyclopropane-1-carboxylic acid (ACC), a biosynthetic precursor of ethylene, has long been proposed to act as a mobile messenger in higher plants. However, little is known about the transport system of ACC. Recently, our genetic characterization of an ACC-resistant mutant with normal ethylene sensitivity revealed that lysine histidine transporter 1 (LHT1) functions as a transporter of ACC. As amino acid transporters might have broad substrate specificity, we hypothesized that other amino acid transporters including *LHT1* paralogs might have the ACC-transporter activity. Here, we took a gain-of-function approach by transgenic complementation of *lht1* mutant with a selected set of amino acid transporters. When we introduced transgene into the *lht1* mutant, the transgenic expression of *LHT2*, but not of *LHT3* or *amino acid permease 5 (AAP5)*, restored the ACC resistance phenotype of the *lht1* mutant. The result provides genetic evidence that some, if not all, amino acid transporters in Arabidopsis can function as ACC transporters. In support, when expressed in *Xenopus laevis* oocytes, both *LHT1* and *LHT2* exhibited ACC-transporting activity, inducing inward current upon addition of ACC. Interestingly, the transgenic expression of *LHT2*, but not of *LHT3* or *AAP5*, could also suppress the early senescence phenotypes of the *lht1* mutant. Taking together, we propose that plants have evolved a multitude of ACC transporters based on amino acid transporters, which would contribute to the differential distribution of ACC under various spatiotemporal contexts.

## Introduction

Transporters of plant hormones play a pivotal role in cell-cell communication, underpinning tissue-specific differentiation and environmental adaptation of higher plants, as exemplified by various defects of hormone-transporter mutants throughout the life cycle (Park et al., 2017; Do et al., 2018). For the past three decades, the molecular genetic analysis combined with transport assay has uncovered various components of plant transporter systems, including principal transporters of each hormone, except brassinosteroid (Abualia et al., 2018). An emerging theme points to the existence of multiple routes for plant hormones transportation. For example, many transporters implicated in the uptake of metabolites/nutrients take part in hormonal transport (Tsay et al., 1993; Huang et al., 1999; Krouk et al., 2010; Kanno et al., 2012). Despite such progress, we are still far from a comprehensive understanding of the components of hormone transport systems and their regulatory mechanisms (Park et al., 2017).

Ethylene, a gaseous plant hormone, controls diverse aspects of the developmental processes including germination, fruit ripening, leaf and flower senescence, and abscission, as well as the environmental adaptation of higher plants (Lin et al., 2009; Bakshi et al., 2015; Dubois et al., 2018). The triple response of ethylene-treated etiolated seedlings, including apical hook exaggeration, inhibition of root growth, and shortened hypocotyl, represents one of the best characterized ethylene responses. It has been instrumentally exploited for genetic identification of key components of ethylene biosynthesis and signaling (Guzman and Ecker, 1990; Ju and Chang, 2015; Merchante and Stepanova, 2017). Since the first identification of ethylene-insensitive *etr1-1* mutant (Bleecker et al., 1988), molecular genetic analyses in *Arabidopsis* have well established how plant perceive and respond to ethylene *via* core signaling module, which comprised five components, including ethylene receptors (ETR1 and its paralogs)-constitutive triple response1-ethylene insensitive 2-EIN3 binding F-Box 1 (EBF1)/EBF2-ethylene insensitive 3 (EIN3)/EIN3-like (Ju and Chang, 2015; Merchante and Stepanova, 2017).

It has been well characterized how plants control the ethylene level. In higher plants, ethylene is biosynthesized from a nonprotein amino acid, 1-aminocyclopropane-1-carboxylic acid (ACC) by ACC oxidase. ACC is synthesized by ACC synthase (ACS) using S-adenosyl methionine, supplied *via* Yang cycle reaction (Boller et al., 1979; Yang and Hoffman, 1984; John et al., 1985). Synthesis of ACC by ACS is the major rate-limiting step of ethylene biosynthesis (Yang and Hoffman, 1984), while under certain circumstances, e.g., fruit ripening, ACC oxidase also contributes to the increase in ethylene level (Nakatsuka et al., 1998; De Paepe et al., 2004; Rudus et al., 2013). As such, ACS activities are under extensive transcriptional and posttranscriptional regulations (Argueso et al., 2007). Besides its biosynthetic regulation, the amount of ACC is also controlled by conjugation and deamination (Van de Poel and Van Der Straeten, 2014; Vanderstraeten and Van Der Straeten, 2017).

In addition to ACC metabolism, short- or long-distance transport of ACC can also contribute to spatiotemporal distribution of ethylene. The long-distance transport of ACC has been suggested by its presence in the vascular system. (Bradford and Yang, 1980; Finlayson et al., 1991; Else et al., 1995; Morris and Larcombe, 1995). Besides long-distance transport, intracellular transport of ACC was also observed in the vacuole (Tophof et al., 1989) and maize mesophyll vacuole (Saftner and Martin, 1993). While the amino acid transporter system has long been implicated to take part in the transport system of ACC (Lurssen, 1981; Saftner and Baker, 1987), the molecular identity of an ACC transporter has been revealed only very recently (Shin et al., 2015). The loss-of-function mutant of *lysine histidine transporter 1* (*LHT1*) in *Arabidopsis* exhibited dose-dependent resistance to exogenous ACC, but not to gaseous ethylene. In the agreement, the mesophyll protoplast of the *lht1* mutants was impaired in the uptake of ^14^C-ACC. The findings that the *lht1* mutant showed a normal ethylene response in the presence of higher concentrations of ACC and it retains, although partial, uptake activity of ^14^C-ACC implicated the presence of additional transporters. Since *LHT1* belongs to a large gene family encoding amino acid transporters, which comprised more than 100 members in *Arabidopsis* (Pratelli and Pilot, 2014; Tegeder and Masclaux-Daubresse, 2018), it is conceivable that transporters other than LHT1 take part in the uptake of ACC. However, there has been no report available about genetic identification of ACC transporters except *LHT1*. Presumably, genetic redundancy, as well as distinct spatiotemporal expression patterns among amino acid transporters, might have hampered the genetic identification of mutants impaired in the uptake of ACC.

To identify additional amino acid transporters that take part in the uptake of ACC, here we undertook a gain-of-function approach making use of the *lht1* mutant, which is defective in the triple response in the presence of 1 µM ACC. After introducing the overexpressing construct of amino acid transporter gene into the *lht1* mutant, we examined whether the defective triple response of *lht1* mutant is restored or not. As a proof of concept, our pilot study with three amino acid transporter genes, namely *LHT2*, *LHT3*, and *AAP5*, revealed that only *LHT2*, but not *LHT3* and *AAP5*, could restore the ACC-induced triple response of the *lht1* mutant. Further, we found that the ectopic expression of *LHT2* could also suppress the early senescence phenotype of the *lht1* mutant. Supporting the hypothesis that LHT2 mediates uptake of ACC, LHT2 exhibited ACC-transporting activity when expressed in *Xenopus* oocytes, as LHT1 did. Together with findings on distinct spatiotemporal expression and substrate specificities of amino acid transporters, we discuss the hypothesis that a multitude of ACC transporting amino acid transporters might have diverse roles during development and environmental adaptation.

## Materials and Methods

### Plant Materials and Growth Conditions

All of the *Arabidopsis thaliana* plant materials used in this study had the “Col-0” ecotype background. The mutants of *lht1-101* (allelic designation was changed hereafter, substituting the original ones, *are2* or *lht1^are2^*), *lht2-1* (SAIL_222_C12), and *lht1-101LHT1ox-1* transgenic plants have been previously described (Shin et al., 2015). The *lht1-101lht2-1* double mutant was obtained by genetic crossing. After crossing *lht1-101* with the *lht2-1* mutant, the resulting F2 population was subject to genotyping PCR to select double mutant plants using the genotyping primers as described (Shin et al., 2015). Unless otherwise stated, seeds were surface sterilized, kept at 4°C for 3 days, and plated on the half strength of MS medium (Duchefa, Haarlem, the Netherlands) supplemented with 0.5% sucrose and 0.7% phytoagar. The seedlings were grown under continuous light. After 7 days, they were transferred to soil (Sunshine Professional Growing Mix #5) for further growth under long days (16 h light/8 h dark) at 22°C. Leaf senescence phenotyping, including leaf yellowing and trypan blue staining, was performed as described (Shin et al., 2015).

### Transgenic Complementation Tests

To examine transgenic complementation of the *lht1-101* mutant, we amplified the full-length cDNA of *LHT2*, *LHT3*, or *AAP5 via* reverse transcriptase–polymerase chain reaction (RT-PCR), using primers listed in **Supplementary Table S1**. The sequence-verified amino acid transporter DNA was subcloned into pENTR1A (Invitrogen, Carlsbad, CA, USA) or pENT-d-TOPO (Invitrogen). The resulting entry clone was recombined into a gateway-compatible plant expression vector, pH2GW7 (Karimi et al., 2002), using LR clonase according to the manufacturer’s instructions (Invitrogen). The pH2GW7/*LHT2*, *LHT3*, or *AAP5* clones were introduced into *Agrobacterium tumefaciens* GV3101 for transformation into the *lht1-101*mutant plants. Putative transgenic plants were selected based on their hygromycin resistance. For phenotypic analysis, homozygous lines harboring a single T-DNA insertion were selected in the T_2_ and T_3_ generations.

### Assays for Seedling Response to ACC or d-Amino Acids

To test the response of seedlings to ACC or d-amino acid, we sowed stratified seeds on plates of MS media containing various concentrations of ACC (Sigma-Aldrich, St. Louis, MO, USA) or of d-amino acids (Sigma-Aldrich). The seedlings were then further grown under continuous light or dark after irradiation for 12 h. For amino acid competition assay with ACC, seedlings were grown on MS media containing 1 μM of ACC with mock or 100 μM of amino acid for 3.5 days in the dark after irradiation of white light for 12 h. The length of hypocotyls and roots was measured using ImageJ software (http://rsbweb.nih.gov/ij). Seedling images were scanned with an Epson scanner (Epson V700 Photo, Epson, Suwa, Nagoya, Japan).

### Expression Analysis

Quantitative real-time PCR (qRT-PCR) analysis was performed with total RNA. After extraction, DNase I–treated total RNA was reverse transcribed by using RevertAid^™^ M-MuLV Reverse Transcriptase (Fermentas, Waltham, MA, USA). The 10-fold diluted cDNA was subject to qRT-PCR analysis using specific primers listed in **Supplementary Table S1**. The qRT-PCR analysis was performed with an Eco^™^ Real-Time PCR System (Illumina, San Diego, CA, USA) using an EvaGreen^®^ qPCR Supermix (Solis Biodyne, Tartu, Estonia). The reactions were performed in technical triplicate for each gene. The comparative ΔCt method was used to evaluate the relative quantities of each amplified product in the samples, as described (Livak and Schmittgen, 2001). Relative expression levels were normalized according to Ct values for *PP2A* (At1g13320), a reference gene (Czechowski et al., 2005).

### *In Vitro* Transcription for cRNA Preparation and Microinjection

The cDNAs encoding *LHT1* or *LHT2* were subcloned into the pGEM-HE vector to be expressed in oocytes using Nde1 restriction enzyme site. After sequence verification, the cRNAs were transcribed from the linearized cDNAs using transcription kit (mMessage mMachine; Ambion, TX, USA) with T7 RNA polymerases. The RNA was dissolved in nucleotide-free water and diluted at a final concentration of 1 μg/μL and then aliquoted and stored at −80°C until use. The nanoinjection of cRNAs (40 ng) into the vegetal or animal pole of each oocyte was carried out using a microinjector (VWR Scientific, Mississauga, Ontario, Canada). The nanoinjector pipettes were pulled out by the glass capillary tubes that were used for the electrodes and polished to a ∼20-μm outer diameter.

### *Xenopus* Oocyte Electrophysiology

The handling of *Xenopus laevis* oocytes and preparation of single cells were described in the previous study (Bai et al., 2019). Briefly, frogs caring procedures followed the Chonnam National University animal caring institution guidelines (CNU IACUC-YB-2016-07, July 2016). The removed oocytes from *X. laevis* were collagenized with shaking for 2 h in Ringer solution (96 mM NaCl, 1 mM MgCl_2_, 2 mM KCl, and 20 mM HEPES at pH 7.5). The matured oocytes were selected and incubated in ND96 containing: 96 mM NaCl, 1 mM MgCl_2_, 2 mM KCl, 1.8 mM CaCl_2_, and 20 mM HEPES at pH 5.6 with 1% penicillin and streptomycin (Sigma). Two electrode voltage clamp experiments were carried out after 48 h for each of the RNA-injected oocytes (Naik, 2019). The oocyte was put in a perfusion chamber (Warner Instrument, Holliston, MA, USA) and flowed with ND96 medium at 1 mL/min. Each oocyte was penetrated with microelectrodes filled up with electrolyte solution. The microelectrodes resistance was from 0.5 to 0.8 MΩ. The electrophysiological experiment was performed at room temperature with oocyte clamp amplifier (OC-726C; Warner Instruments) and acquisition of data was performed using Digidata 1320 and pClamp 9 (Molecular Devices, San Jose, CA, USA).

## Results

**Figure 1:**
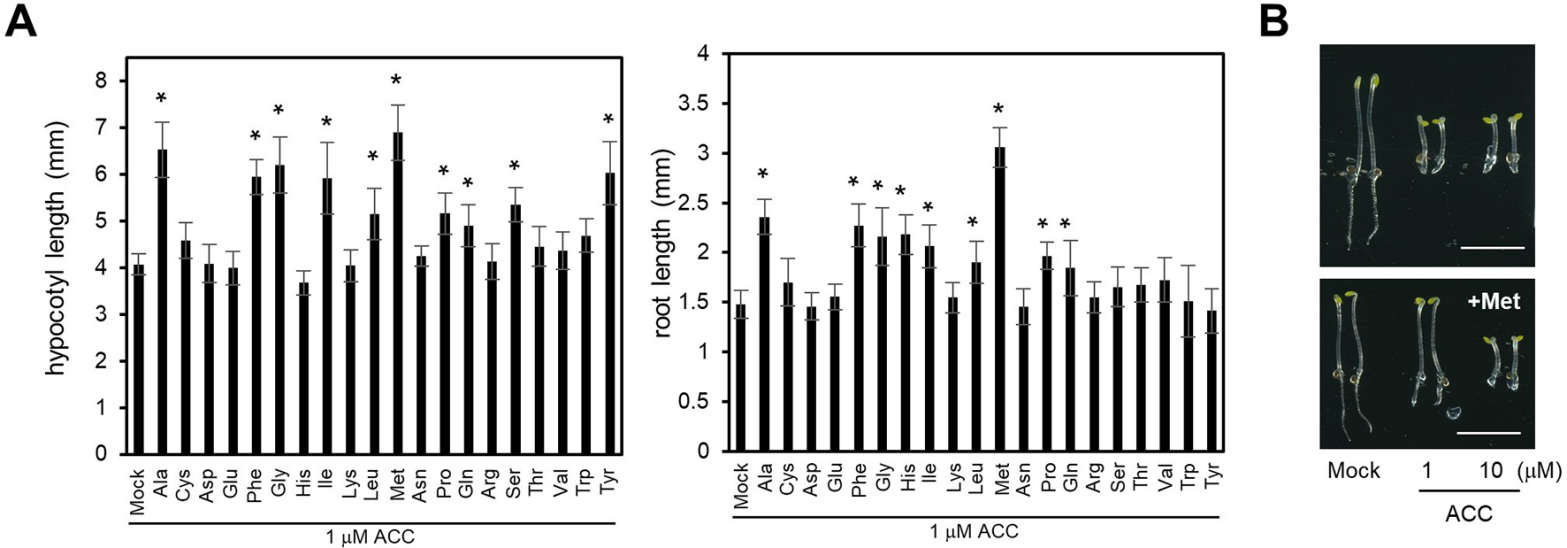
Effect of supplemented amino acids on ACC-induced inhibition of hypocotyl and root growth. **(A)** Hypocotyl (left) and root length (right). The wild-type seedlings were grown under dark for 4 days on MS-sucrose media containing 1 µM of ACC supplemented with each of 20 types of 100 µM amino acid. Values are mean ± SD (n = 15). The significant difference of the length between seedlings grown on the media containing ACC only and ACC with amino acid is indicated with asterisks (*t* test, *p < 0.01). **(B)** Representative wild-type seedlings grown on MS-sucrose media containing either 1 or 10 µM of ACC supplemented without (upper) or with 100 µM of methionine (Met) (lower). Scale bar, 5 mm.

Previously we showed that LHT1 takes part in the uptake of ACC in *Arabidopsis* (Shin et al., 2015). However, the null alleles of *LHT1* retained, despite reduced, ACC uptake activity, suggesting that LHT1 may not be the sole ACC transporter in *Arabidopsis*. In line with those findings, when applied at high concentration, various amino acids reduced the ACC-induced triple response (**Figure 1**). We examined the triple response of etiolated seedlings that were grown in the presence of 1 µM ACC plus mock solution or 100 µM of each amino acid. The result showed that 8 of 20 amino acids, including alanine, phenylalanine, glycine, isoleucine, leucine, methionine, glutamine, and proline, alleviated the ACC-induced triple response in dark-grown wild-type seedlings. As shown in **Figure 1A**, the hypocotyl length or root length of seedlings that were grown in the presence of ACC plus above stated amino acids was longer than that of seedling grown in the presence of ACC alone. Some amino acids affected only either hypocotyl growth or root growth. For example, the basic amino acid, histidine, reduced the ACC-induced root growth inhibition, but not hypocotyl growth. In the case of serine and tyrosine, these affected only hypocotyl growth (**Figure 1A**). On the contrary, higher concentration of ACC (10 µM) could overcome the inhibitory effect of the amino acid, e.g., methionine, inducing the characteristic triple response: inhibition of hypocotyl elongation, shortened root growth, and exaggerated apical hook formation (**Figure 1B**). These results implied that some, if not all, amino acids may compete with ACC for uptake. Further, the diverse profile of amino acids with inhibitory activity for ACC-induced triple response suggested the presence of a multitude of amino acid transporters with ACC-transporting activity in *Arabidopsis*.

**Figure 2:**
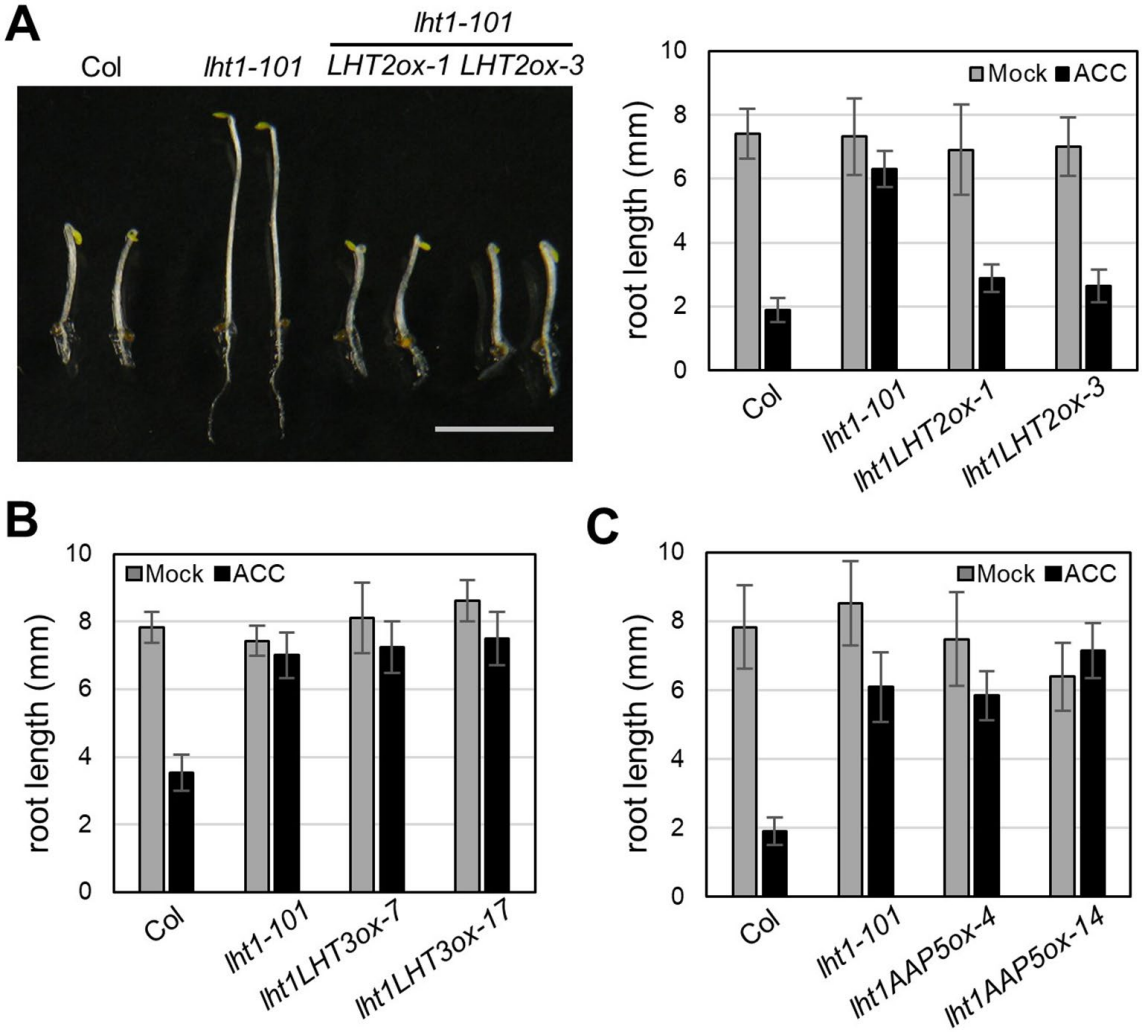
Transgenic complementation of *lht1-101* by overexpression of *LHT2, LHT3*, or *AAP5*. **(A)** Restored ACC response of *lht1-101* mutant by overexpression of *LHT2*. Left, Morphology of representative 4-day-old seedlings grown on MS-sucrose under dark media in the presence of 1 µM ACC. Note that the triple response of *lht1-101* mutant was restored in the two independent *LHT2*-overexpressing transgenic lines (*LHT2ox1* and *LHT2ox3*). Scale bar, 5 mm. Right, Root length of the transgenic *lht1-101* seedlings. The seedlings were grown on MS-sucrose media supplemented with either Mock or 1 µM of ACC for 4 days under dark. Values are mean ± SD (n = 15). **(B)** Root length of *LHT3* overexpressing transgenic *lht1-101* seedlings. The seedlings were grown as described in **(A)**. Values are mean ± SD (n = 15). **(C)** Root length of *AAP5* overexpressing transgenic *lht1-101* seedlings. Values are mean ± SD (n = 15). The seedlings were grown as described in **(A)**.

**Figure 3:**
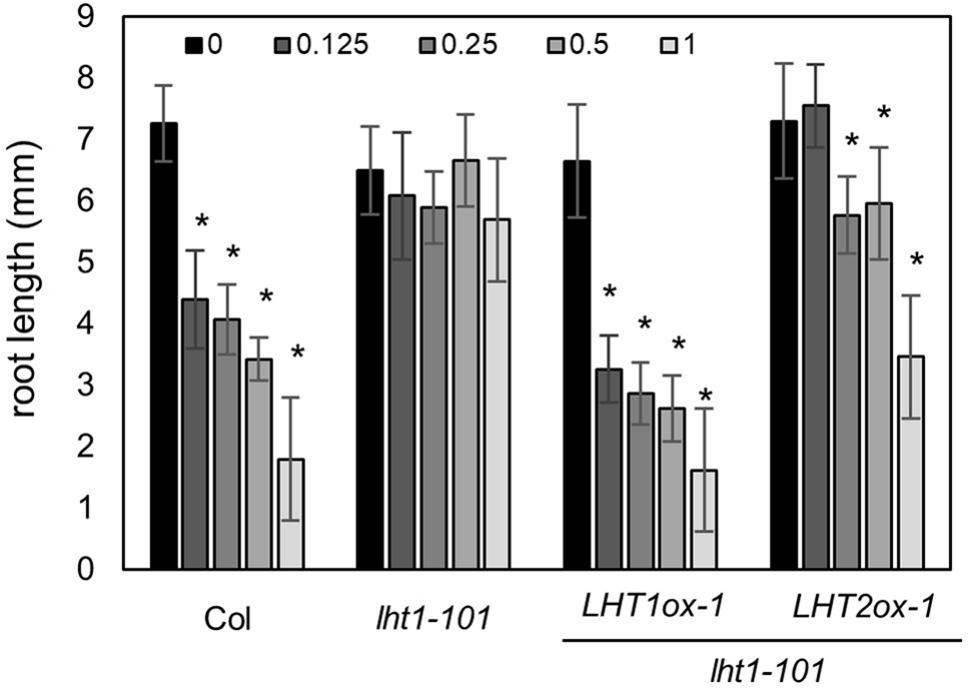
ACC dose-response analysis of transgenic *lht1-101* seedlings overexpressing *LHT2*. Wild-type, *lht1-101*, the transgenic *lht1-101* seeds overexpressing *LHT1* or *LHT2* were grown on MS-sucrose media containing indicated concentrations (µM) of ACC. The seedlings were grown for 4 days in the dark. Values are mean ± SD (n = 15). A significant difference between ACC treated and mock-treated (0 ACC) seedlings was indicated with asterisks (*t* test, *p* < 0.01).

Although *LHT1* was identified as ACC transporter using the loss-of-function approach, all knockout mutants of *LHT* family gene except *lht1* did not show ACC resistance in seedlings (Shin et al., 2015). Because the phenotype of knockout mutants is highly dependent on the developmental stage due to the specific spatiotemporal expression of the genes (**Supplementary Figure 1**) and can be masked by other gene activity with overlapping function, the possibility that other LHTs are involved in ACC transport cannot be ruled out. To overcome the drawbacks of the loss-of-function approach, we adopted a transgenic gain-of-function approach to identify ACC-transporting amino acid transporters. After introducing transgene that overexpresses a gene encoding amino acid transporter into the *lht1-101* mutant, a null allele, we tested if it restored the defect in the ACC-induced triple response of the *lht1* mutant. As a proof of concept, we selected three amino acid transporters (LHT2, LHT3, and AAP5) in two amino acid transporter family, e.g., LHT and AAP family. When the transgene was introduced into the *lht1* mutant, the resulting 17 of 19 T2 lines with *35S*::LHT2 showed normal triple response to ACC as wild type did. In contrast, none of more than 20 T2 lines with *35S*::LHT3 or *35S*:AAP5 exhibited defective triple response to ACC as the *lht1-101* mutant did. After confirmation of the elevated transcript level of each amino acid transporter (**Supplementary Figure 2**), we examined the ACC-induced triple response in detail with two independent homozygous lines that overexpressed each amino acid transporter (**Figure 2**). In the presence of 1 μM of ACC, wild type showed typical triple response including apical hook exaggeration, inhibition of hypocotyl growth, and shortened root growth, whereas *lht1-101* mutant did not (**Figure 2A**). As shown in **Figures 2B, C** overexpression of *LHT2*, but not *LHT3* or *AAP5*, could restore the ACC-induced triple response of the *lht1-101* mutant. Next, we analyzed the concentration-dependent ACC response in the *lht1-101* mutant overexpressing *LHT1* or *LHT2*. *LHT1* overexpression in the *lht1-101* mutant restored the ACC response of root growth inhibition completely as wild type did at all concentrations of ACC tested, whereas in transgenic *lht1-101* seedlings overexpressing *LHT2*, the ACC induced root growth inhibition was observed at high concentration of ACC (**Figure 3**). Despite the lack of any visible effect of the *lht2-1* mutation on the ACC-induced triple response (**Supplementary Figure 3**), our results of transgenic complementation demonstrated the role of LHT2 as a potential ACC transporter, functioning likewise LHT1.

**Figure 4:**
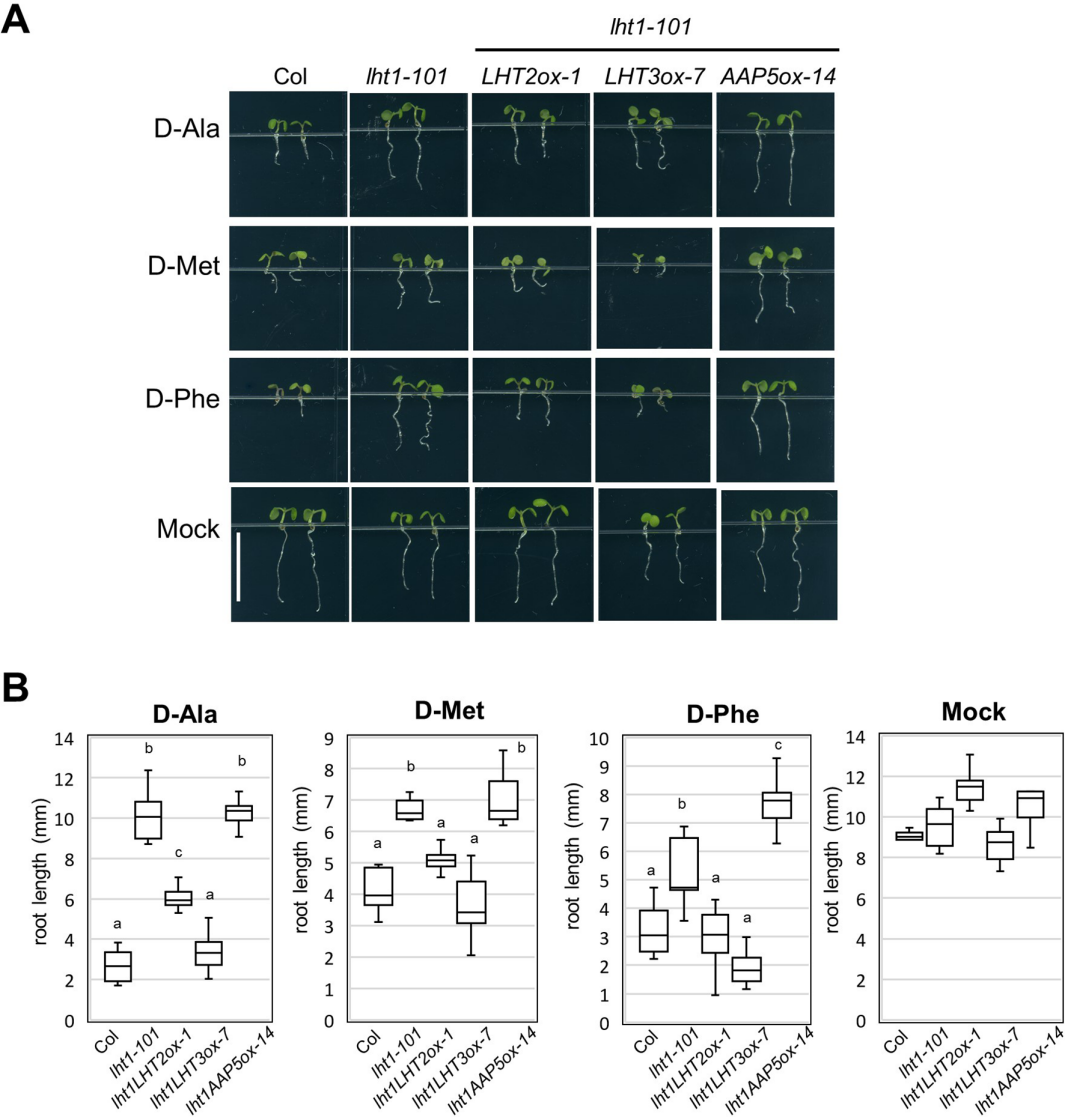
d-Amino acid response of transgenic *lht1-101* seedlings overexpressing amino acid transporters. **(A)** Morphology of representative seedlings that have grown on MS-sucrose media containing mock, 1 mM of d-alanine (d-Ala), 3 mM of d-methionine (d-Met), or 3 mM of d-phenylalanine (d-Phe). Wild-type, *lht1-101*, and the transgenic *lht1-101* plants overexpressing the amino acid transporter, e.g., *LHT2*, *LHT3*, or *AAP5*, were grown for 5 days under continuous light. Scale bar, 5 mm. **(B)** The root length of the seedlings grown as described in **(A)**. The data are presented with box-and-whisker plot (n = 6-11). Different letters indicate significant differences at *p* < 0.01 according to one-way analysis of variance with Tukey honestly significant difference test. Evidence for normality of distribution and homoscedasticity is presented in **Supplementary Table S2**.

LHT1 mediates uptake of broad substrates, including several d-amino acids, illustrated by the strong resistant phenotype of the *lht1* mutant in the presence of a high concentration of d-amino acids (Svennerstam et al., 2007; Gordes et al., 2011). With the assumption that other amino acid transporters take part in the uptake of the d-amino acids, substrates of LHT1, we investigated whether d-amino acid resistance of the *lht1* mutant could be restored by transgenic overexpression of *LHT2*, *LHT3*, or *AAP5*. The results showed that overexpression of *LHT2*, or *LHT3*, but not of *AAP5*, restored the sensitivity to d-amino acids, including d-Ala, d-Phe, and d-Met in *lht1-101* (**Figure 4**). Together with the finding that *LHT3* could not restore ACC resistance of the *lht1* mutant (**Figure 2**), these results implied that LHT3 might have similar but distinct substrate specificity with LHT1 and LHT2.

**Figure 5:**
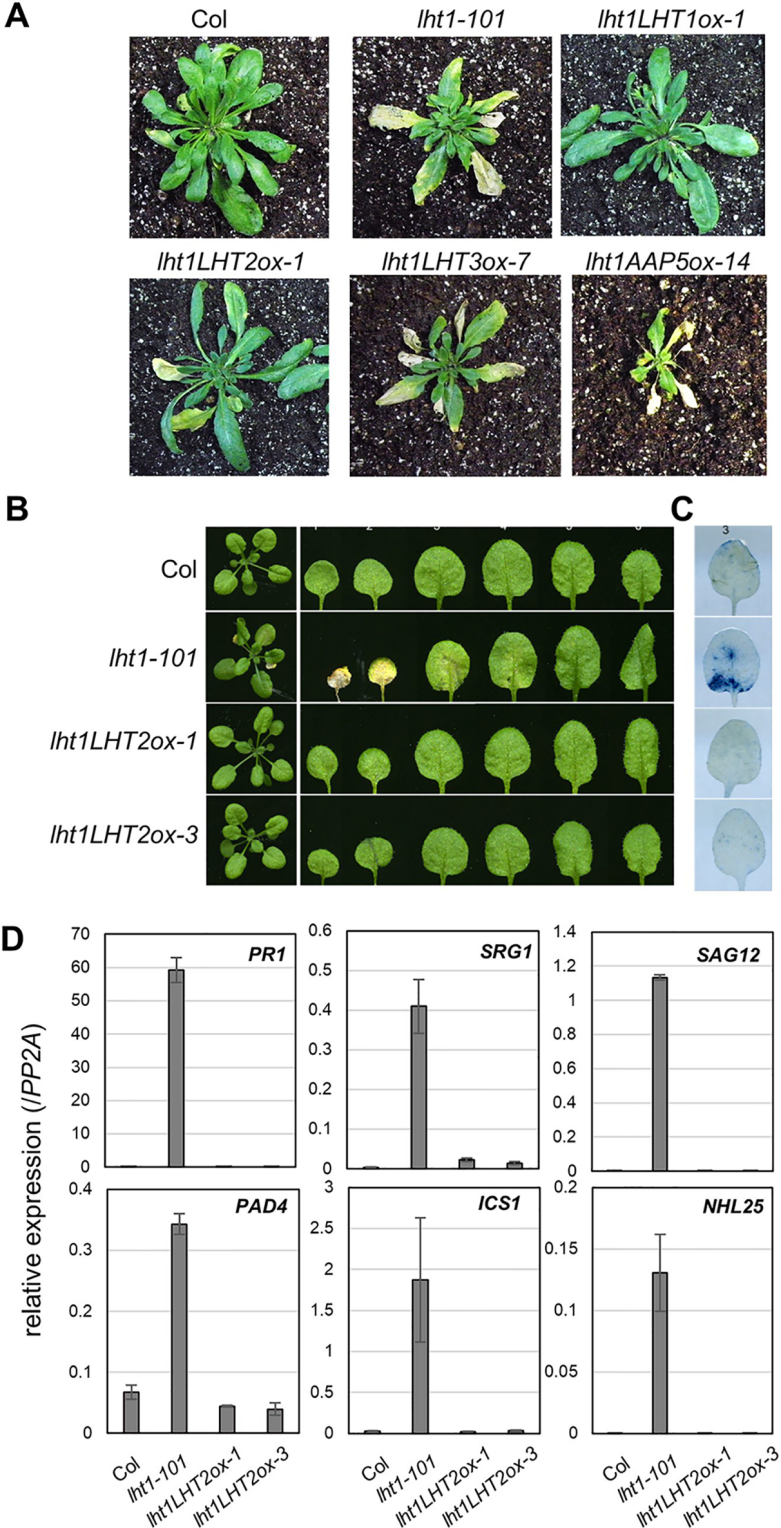
The early senescence syndrome of the transgenic *lht1-101* mutant overexpressing *LHT2*. **(A)** Representative morphology of wild-type, *lht1-101*, and the transgenic plants overexpressing *LHT2, LHT3*, or *AAP5* at 38 DAG (days after growth). For clarity, the inflorescence of each plant was removed. **(B)** Representative leaf morphology of the transgenic *lht1-101* plants overexpressing *LHT2* at 27 DAG. **(C)** Trypan blue staining. Cell death in the third leaf of each plant pictured in **(B)** was assayed with trypan blue. **(D)** Expression analysis of senescence-associated genes. The fourth leaves of the plants at 27 DAG were sampled for extraction of total RNA, which was subjected to qRT-PCR analysis. For each gene indicated, the relative expression was presented after normalization with the level of *PP2A*. Values are mean ± SD (n = 3, technical triplicate).

Early senescence/cell death syndrome is a characteristic developmental phenotype of the *lht1* mutants (Liu et al., 2010; Shin et al., 2015). We investigated whether the early senescence phenotype of *lht1-101* could be altered in the transgenic plants overexpressing the amino acid transporters. Interestingly, overexpression of *LHT2* restored the accelerated leaf yellowing phenotype, an early leaf senescence syndrome of *lht1* mutants, while overexpression of *LHT3* or *AAP5* did not (**Figure 5A**). When we performed a closer examination of *LHT2* overexpressing *lht1-101* plants, we found that *LHT2* overexpression suppressed the aberrant cell-death phenotype of the *lht1-101* mutant, manifested by trypan blue staining (**Figures 5B, C**). As the early senescence phenotype of the *lht1* mutant is accompanied by the massive changes of gene expression (Liu et al., 2010), further we tested whether overexpression of *LHT2* can restore the elevated expression of a set of senescence-associated genes, including *pathogenesis related 1* (*PR1*), *senescence related gene 1* (*SRG1*), *senescence associated gene 12* (*SAG12*), *phytoalexin deficient 4* (*PAD4*), *isochorismate synthase 1* (*ICS1*), *NDR1/HIN1-like 25* (*NHL25*) in the *lht1-101* mutant (**Figure 5D**). The expression of senescence-associated genes in the *lht1-101* was shown to be suppressed by transgenic *LHT2* overexpression. Collectively, these results implied that the ectopically expressed *LHT2* can substitute the role of *LHT1* for the control of leaf senescence.

**Figure 6:**
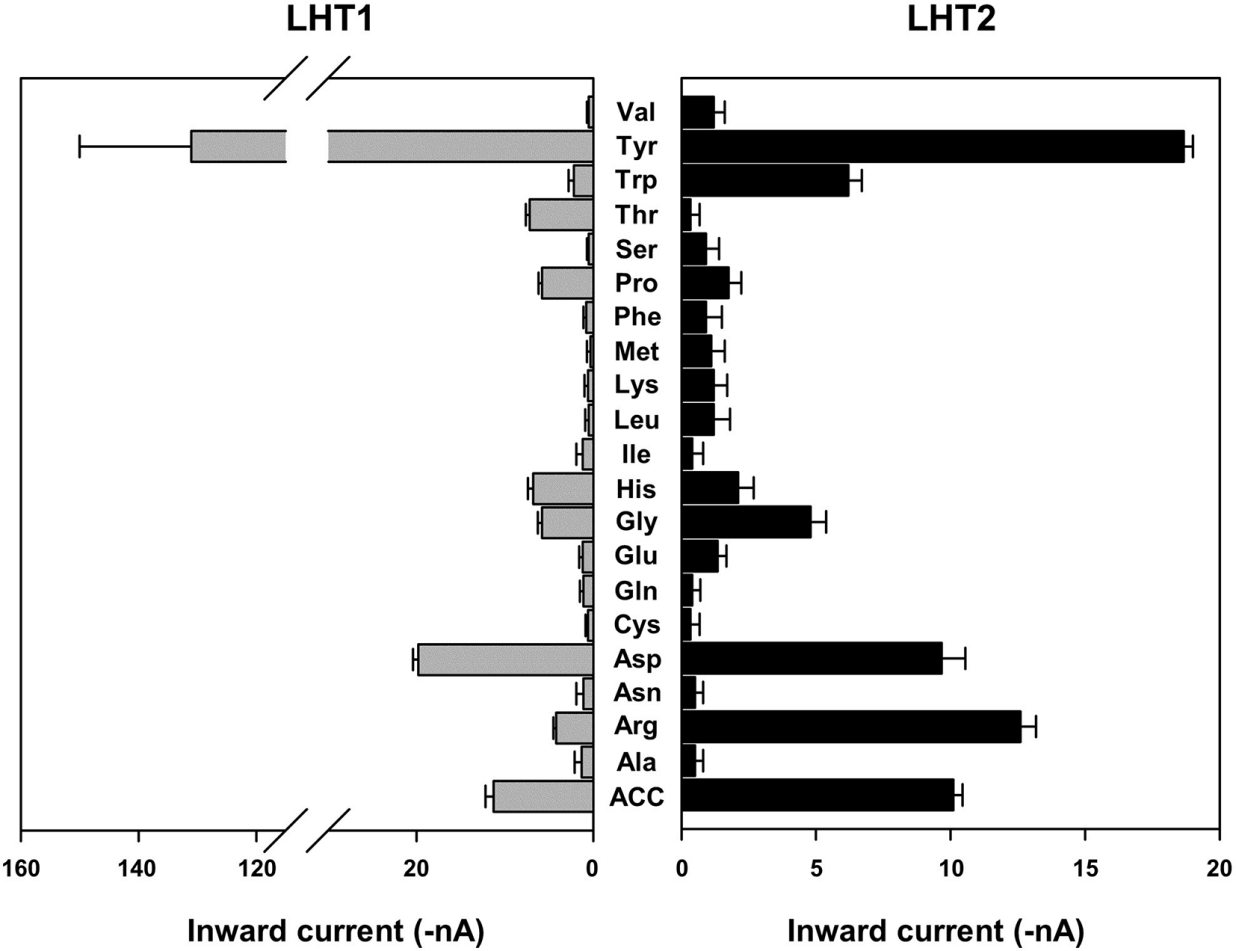
Amino acid selectivity of LHT1 and LHT2 in *Xenopus* oocytes. After perfusion with Ringer solution at pH 5.6, oocytes expressing *LHT1* or *LHT2* were treated with each amino acid (100 mM) or ACC (100 mM). For tyrosine, the currents were measured at 2.5 mM (limit of solubility). The resulting inward currents were recorded at −80 mV. Data represent the means ± SEM (n = 6–8 oocytes/four different frogs).

**Figure 7:**
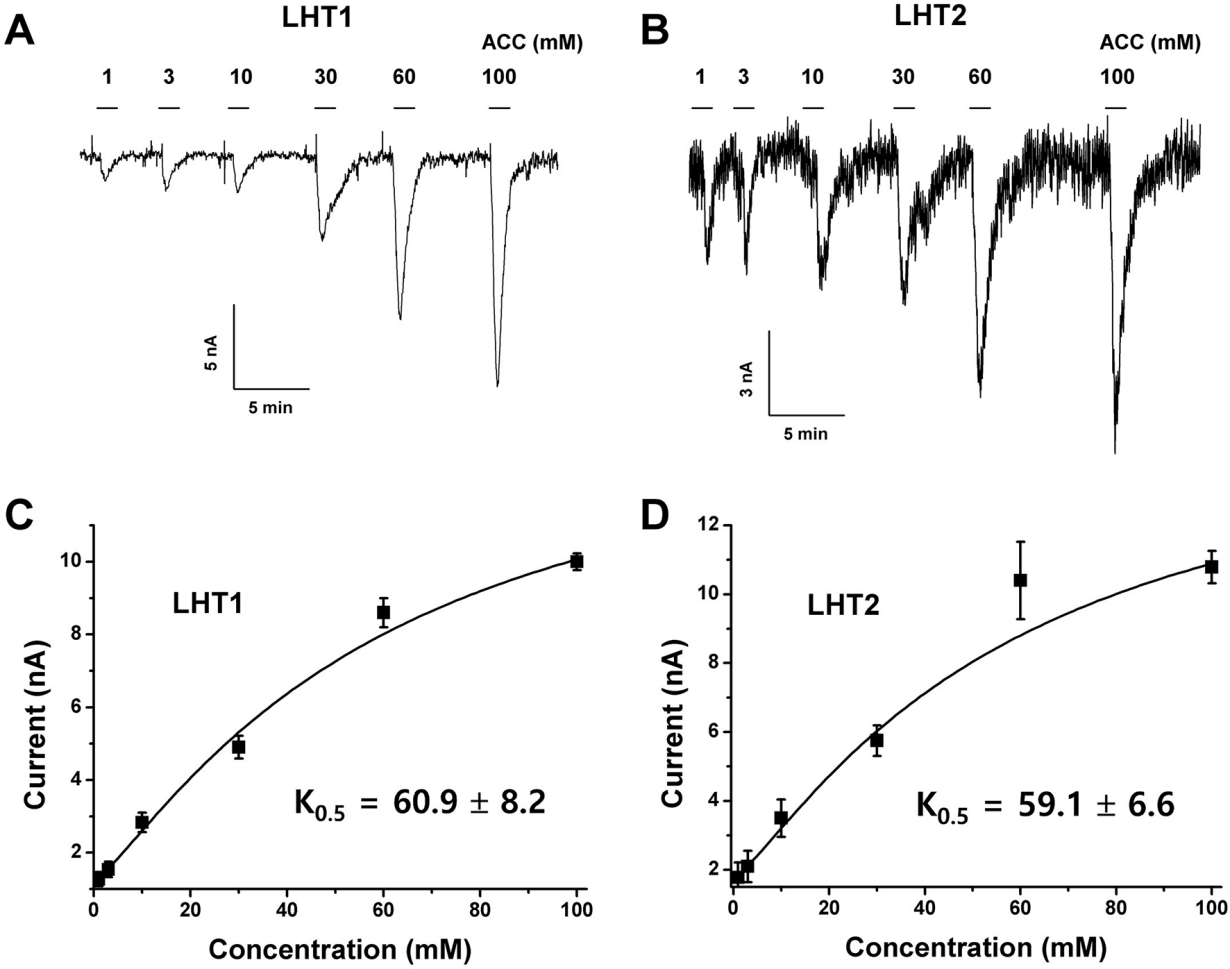
Concentration dependency and kinetic analysis of ACC-induced inward currents in *LHT*-expressing *Xenopus* oocytes. **(A** and **B)** The representative traces of *LHT1*- or *LHT2*-expressing oocytes after addition of various concentration of ACC. Exogenously applied ACC produced a reversible inward current. The holding potential was −80 mV. ACC induced the inward currents with the concentration-dependent manner in each *LHT*-expressing oocyte, respectively. **(C** and **D)** Kinetic analysis of ACC-induced inward currents. *K*_0.5_ value was calculated with Michaelis-Menten’s equation. Data represent the means ± SE.M (n = 6–8 oocytes/four different frogs).

Given the genetic evidence that LHT1 and LHT2 play similar roles as an ACC/amino acid transporter, we assessed the biochemical activity of LHT1 and LHT2. Taking an electrophysiological approach based on *Xenopus* oocyte system, we conducted a two-electrode voltage-clamp electrophysiological recording. After injection into *Xenopus* oocytes with mRNA of *LHT1* or *LHT2*, we measured inward currents elicited after the addition of the substrate, ACC or amino acids. The applied ACC or amino acids (100 mM) did not induce any current in the control oocytes (**Supplementary Figure 4**). In contrast, the treatment of a series of amino acids induced inward currents with differential activity in both *LHT1*- and *LHT2*-expressing oocytes (**Figure 6**). Notably, LHT1 and LHT2 exhibited largely similar, if not identical, substrate selectivity. The results implied that LHT1 and LHT2 transport overlapping set of amino acid substrates, but with differential preference. Under the same condition, exogenously applied ACC elicited the inward currents on both *LHT1*- and *LHT2*-expressing oocytes, confirming their roles as ACC transporters (**Figures 6** and **7**). Further, we evaluated the ACC dose dependency in the *LHT1*- or *LHT2*-expressing oocytes. With the dose-response analysis, we calculated *K*_0.5_ for ACC with nonlinear regression using Michaelis-Menten’s equation. The *K*_0.5_ values of ACC are 60.9 ± 8.2 mM and 59.1 ± 6.6 mM for *LHT1*- and *LHT2*-expressing oocytes, respectively, suggesting that LHT1 and LHT2 can function as ACC transporter with comparable activity in *Xenopus* oocytes.

## Discussion

As a precursor of ethylene, ACC has long been recognized as a mobile molecule in higher plants (Bradford and Yang, 1980; Amrhein et al., 1982; Métraux and Kende, 1983; Else et al., 1995; English et al., 1995; Kende et al., 1998; Vriezen et al., 2003). In accordance, intercellular ACC transport has been suggested to contribute to the ethylene-mediated stress adaptation and development (Woltering, 1990; Jones and Woodson, 1997; Jones and Woodson, 1999; Dugardeyn et al., 2008; Vanderstraeten and Van Der Straeten, 2017). However, the molecular nature of ACC-transporting system has remained elusive until recent genetic identification of *lht1-101*, as an ACC-resistant mutant (Shin et al., 2015). In line with previous biochemical studies (Lurssen, 1981; Saftner and Baker, 1987), we found that a series of amino acids could inhibit the ACC-induced triple response, suggestive of the presence of a multitude of ACC transporters (**Figure 1**). However, it should be taken into account that the inhibitory effects of amino acids may not necessarily involve their competitive effect for ACC uptake, considering that certain amino acids may alter hormonal biosynthesis or cellular signaling components (Qi et al., 2006; Luna et al., 2014; Kong et al., 2016; Smith et al., 2017). Other ACC transporters than LHT1 have also been inferred by the physiological features of the *lht1* mutant, including its partial ACC resistance as well as a partial defect in ACC uptake activity. Still, it is challenging to identify the ACC-transporting amino acid transporters among more than 100 putative amino acid transporters in *Arabidopsis* (Tegeder and Masclaux-Daubresse, 2018).

In this study, we presented that a gain-of-function approach, transgenic complementation of the *lht1* mutant, is a feasible way to identify ACC transporters *in planta*. As a proof of concept, our pilot study revealed that LHT2 can function as an ACC transporter. When overexpressed in the *lht1* mutant background, *LHT2* could restore all of the physiological defects of the *lht1* mutant, including defective ACC-induced triple response, d-amino acid resistance, and early-senescence syndromes (**Figures 2**–**5**). Although we cannot rule out the possibility that the overexpression of *LHT2* may trigger the expression other ACC-transporting amino acid transporters to complement the *lht1* mutant phenotype, we found that the oocytes expressing *LHT1* or *LHT2* were responsive to exogenous ACC, producing inward currents with comparable efficacy (**Figure 7**). The results demonstrate that LHT1/LHT2 act as an ACC transporter in *Xenopus* oocytes at millimolar concentrations of ACC. It is noteworthy that the *K*_0.5_ values of ACC for LHT1 or LHT2 were higher than expected. Considering that 1 µM of ACC is high enough to elicit the full range of the triple response (**Figure 2**), it seems that our experimental conditions may not be optimal for the transport assay of LHT1 or LHT2 proteins. Alternatively, but not mutually exclusively, LHT1 or LHT2 proteins may require additional plant protein(s) or posttranslational modification for their full activity. Thus, it remains to be proven that LHT2 functions as an ACC transporter at physiologically relevant concentrations *in planta*. While both LHT1 and LHT2 could rescue the ACC-resistant phenotype of *lht1* mutant, we observed slight difference in the ACC dose-response phenotypes among the wild-type and transgenic lines of LHT1 and LHT2 (**Figure 3**). Compared to *LHT1*-overexpressing line, the plants with overexpression of *LHT2* were less sensitive to exogenously applied ACC. It may be due to relatively lower transporter activity of LHT2 compared to LHT1 *in planta*. However, as we did not assess the protein level of LHT1 and LHT2, it cannot be ruled out that protein expression/stability underlies the differential dose-response phenotypes.

On the contrary, *LHT3* and *AAP5* were not effective in the complementation activity of the *lht1* mutant (**Figures 2**–**5**), suggesting that these amino acid transporters have marginal, if any, ACC-transporting activity. In agreement, compared to LHT1 or LHT2 (**Figure 6**), AAP5 has been characterized to have differential substrate selectivity, mediating uptake of basic amino acids (Boorer and Fischer, 1997; Fischer et al., 2002), while the substrate of LHT3 has not been reported yet. It is intriguing that transgenic expression of *LHT3* could restore the resistance of the *lht1* mutant to d-Ala, d-Met, and d-Phe, implicating that LHT3 might have overlapping activity with LHT1 toward a subset of LHT1 substrates, except ACC. Notably, the ACC-transporting LHT1 and LHT2 exhibited similar, but not identical, amino acid selectivity in the oocyte system (**Figure 6**). These results imply that amino acid transporter have delicate transporting channels, selecting specific substrates. It is noteworthy that methionine was not the preferred substrate for LHT1 or LHT2 in the oocyte system (**Figure 6**), although it is one of the most effective amino acids in the ACC-competition assay (**Figure 1**; Lurssen, 1981). Taken together, it is plausible that other class of amino acid transporters with differential substrate selectivity may also take part in the uptake of ACC, along with LHT1/LHT2.

*LHT2* was previously reported to be expressed preferentially in floral organs, mainly in tapetum tissues and pollens (**Supplementary Figure 1**) (Lee and Tegeder, 2004). The tissue-preferential expression of *LHT2* may account for the lack of defect of *lht2-1* mutant seedlings in regard to the ACC-induced triple response (**Supplementary Figure 3**). For the present, it is not clear whether the ACC-transporting activity of LHT2 has any physiological relevance during reproduction. It is noteworthy that high-order, octuple, *ACS* mutants of *Arabidopsis* exhibited pleiotropic defects unlike mutants with impaired ethylene signaling, including reduced fertility (Tsuchisaka et al., 2009). As we did not observe any reproductive defects in the *lht2-1* mutant and several *LHT* genes are also expressed in pollens (**Supplementary Figure 1**), it is possible that there might be other pollen-expressed, ACC-transporting amino acid transporters, functioning redundantly with LHT2. It will be interesting to examine whether the pollen-expressed *LHT*s have similar substrate selectivity and whether multiple loss-of-function *lht* mutants bear any defects in reproductive processes.

It is intriguing that *LHT2*, but not *LHT3* or *AAP5*, could complement the early senescence syndrome of the *lht1* mutant, which entails transcriptional alteration of several defense/cell death–related genes (**Figure 5**). Although it has not been resolved how *lht1* mutation impacts on leaf senescence and cell death syndrome (Liu et al., 2010; Shin et al., 2015), it is tempting to speculate that ACC or possibly other shared substrates of LHT1 and LHT2 play signaling functions in these processes. Together with accumulating evidence that proteogenic and nonproteogenic amino acids have diverse regulatory roles (Qi et al., 2006; Luna et al., 2014; Ramesh et al., 2015; Kong et al., 2016; Dinkeloo et al., 2018; Shi et al., 2018), future studies about how those amino acid transporters modulate leaf senescence would shed light on novel functions of ACC or amino acids during leaf senescence/cell death syndrome.

Together with electrophysiological analysis, the transgenic complementation analysis of *lht1* mutant would facilitate further characterization of amino acid transporter genes, expanding the repertoire of ACC-transporting amino acid transporters. As ACC has been recently appreciated as a signaling molecule (Xu et al., 2008; Tsuchisaka et al., 2009; Tsang et al., 2011; Yoon and Kieber, 2013), the identification of ACC-transporting amino acid transporters along with their spatiotemporal expression specificity would help to elucidate the role of ACC and amino acid transporters during development and environmental adaptation in higher plants.

## Data Availability

All datasets [Expression Analysis] for this study are included in the manuscript and the **Supplementary Files**.

## Author Contributions

M-SS and SL designed the research. JC, SE, KS, RL, SC, and SL performed the research. KS, JL, SL, and M-SS analyzed the data. SL, JL and M-SS wrote the paper.

## Funding

This work was supported by Basic Science Research Program through the National Research Foundation of Korea funded by the Ministry of Education (2018R1D1A1B07049914, 2019R1F1A1059156, and 2019R1I1A1A01044043), the Rural Development Administration (Next-Generation Biogreen21 program [Agricultural Biotechnology Research Center no. PJ01369001]), and by the Ministry of Science, ICT & Future Planning (2015R1C1A2A01052950).

## Conflict of Interest Statement

The authors declare that the research was conducted in the absence of any commercial or financial relationships that could be construed as a potential conflict of interest.

## References

[B1] Abualia R. Benkova E. Lacombe B. (2018). “Chapter five—transporters and mechanisms of hormone transport in *Arabidopsis*,” in Advances in botanical research. Ed. Maurel C. (Academic Press), 115–138. 10.1016/bs.abr.2018.09.007

[B2] Amrhein N. Breuing F. Eberle J. Skorupka H. Tophof S. (1982) "The metabolism of 1-aminocyclopropane-1-carboxylic acid." in Plant Growth Substances.Ed. Wareing P. F. (London, UK: Academic Press), 249–258.

[B3] Argueso C. Hansen M. Kieber J. (2007). Regulation of ethylene biosynthesis. J. Plant Growth Regul. 26 (2), 92–105. 10.1007/s00344-007-0013-5

[B4] Bai H. W. Eom S. Yeom H. D. Nguyen K. V. A. Lee J. Sohn S. O. (2019). Molecular basis involved in the blocking effect of antidepressant metergoline on C-type inactivation of Kv1.4 channel. Neuropharmacology 146, 65–73. 10.1016/j.neuropharm.2018.11.024 30465811

[B5] Bakshi A. Shemansky J. M. Chang C. R. Binder B. M. (2015). History of research on the plant hormone ethylene. J. Plant Growth Regul. 34 (4), 809–827. 10.1007/s00344-015-9522-9

[B6] Bleecker A. B. Estelle M. A. Somerville C. Kende H. (1988). Insensitivity to ethylene conferred by a dominant mutation in *Arabidopsis thaliana*. Science 241 (4869), 1086–1089. 10.1126/science.241.4869.1086 17747490

[B7] Boller T. Herner R. C. Kende H. (1979). Assay for and enzymatic formation of an ethylene precursor, 1-aminocyclopropane-1-carboxylic acid. Planta 145 (3), 293–303. 10.1007/BF00454455 24317737

[B8] Boorer K. J. Fischer W. N. (1997). Specificity and stoichiometry of the *Arabidopsis* H+/amino acid transporter AAP5. J. Biol. Chem. 272 (20), 13040–13046. 10.1074/jbc.272.20.13040 9148914

[B9] Bradford K. J. Yang S. F. (1980). Xylem transport of 1-aminocyclopropane-1-carboxylic acid, an ethylene precursor, in waterlogged tomato plants. Plant Physiol. 65 (2), 322–326. 10.1104/pp.65.2.322 16661182 PMC440319

[B10] Czechowski T. Stitt M. Altmann T. Udvardi M. K. Scheible W.-R. (2005). Genome-wide identification and testing of superior reference genes for transcript normalization in *Arabidopsis*. Plant Physiol. 139 (1), 5–17. 10.1104/pp.105.063743 16166256 PMC1203353

[B11] De Paepe A. Vuylsteke M. Van Hummelen P. Zabeau M. Van Der Straeten D. (2004). Transcriptional profiling by cDNA-AFLP and microarray analysis reveals novel insights into the early response to ethylene in *Arabidopsis*. Plant J. 39 (4), 537–559. 10.1111/j.1365-313X.2004.02156.x 15272873

[B12] Dinkeloo K. Boyd S. Pilot G. (2018). Update on amino acid transporter functions and on possible amino acid sensing mechanisms in plants. Semin. Cell Dev. Biol. 74, 105–113. 10.1016/j.semcdb.2017.07.010 28705659

[B13] Do T. H. T. Martinoia E. Lee Y. (2018). Functions of ABC transporters in plant growth and development. Curr. Opin. Plant Biol. 41, 32–38. 10.1016/j.pbi.2017.08.003 28854397

[B14] Dubois M. Van den Broeck L. Inzé D. (2018). The pivotal role of ethylene in plant growth. Trends Plant Sci. 23 (4), 311–323. 10.1016/j.tplants.2018.01.003 29428350 PMC5890734

[B15] Dugardeyn J. Vandenbussche F. Van Der Straeten D. (2008). To grow or not to grow: what can we learn on ethylene-gibberellin cross-talk by in silico gene expression analysis? J. Exp. Bot. 59 (1), 1–16. 10.1093/jxb/erm349 18212030

[B16] Else M. A. Hall K. C. Arnold G. M. Davies W. J. Jackson M. B. (1995). Export of abscisic acid, 1-aminocyclopropane-1-carboxylic acid, phosphate, and nitrate from roots to shoots of flooded tomato plants (accounting for effects of xylem sap flow rate on concentration and delivery). Plant Physiol. 107 (2), 377–384. 10.1104/pp.107.2.377 12228364 PMC157137

[B17] English P. J. Lycett G. W. Roberts J. A. Jackson M. B. (1995). Increased 1-aminocyclopropane-1-carboxylic acid oxidase activity in shoots of flooded tomato plants raises ethylene production to physiologically active levels. Plant Physiol. 109 (4), 1435–1440. 10.1104/pp.109.4.1435 12228680 PMC157679

[B18] Finlayson S. A. Foster K. R. Reid D. M. (1991). Transport and metabolism of 1-aminocyclopropane-1-carboxylic acid in sunflower (*Helianthus annuus* L.) seedlings. Plant Physiol. 96 (4), 1360–1367. 10.1104/pp.96.4.1360 16668342 PMC1080938

[B19] Fischer W. N. Loo D. D. Koch W. Ludewig U. Boorer K. J. Tegeder M. (2002). Low and high affinity amino acid H+-cotransporters for cellular import of neutral and charged amino acids. Plant J. 29 (6), 717–731. 10.1046/j.1365-313X.2002.01248.x 12148530

[B20] Gordes D. Kolukisaoglu U. Thurow K. (2011). Uptake and conversion of d-amino acids in *Arabidopsis thaliana*. Amino Acids 40 (2), 553–563. 10.1007/s00726-010-0674-4 20593294

[B21] Guzman P. Ecker J. R. (1990). Exploiting the triple response of *Arabidopsis* to identify ethylene-related mutants. Plant Cell 2 (6), 513–523. 10.1105/tpc.2.6.513 2152173 PMC159907

[B22] Huang N.-C. Liu K.-H. Lo H.-J. Tsay Y.-F. (1999). Cloning and functional characterization of an *Arabidopsis* nitrate transporter gene that encodes a constitutive component of low-affinity uptake. Plant Cell 11 (8), 1381–1392. 10.1105/tpc.11.8.1381 10449574 PMC144300

[B23] John P. J.r A. Miller P. Miller A. J. (1985). Activity of the ethylene-forming enzyme measured *in vivo* at different cell potentials. J. Plant Physiol. 121 (5), 397–406. 10.1016/S0176-1617(85)80076-6

[B24] Jones M. L. Woodson W. R. (1997). Pollination-induced ethylene in carnation (role of stylar ethylene in corolla senescence). Plant Physiol. 115 (1), 205–212. 10.1104/pp.115.1.205 12223801 PMC158476

[B25] Jones M. L. Woodson W. R. (1999). Differential expression of three members of the 1-aminocyclopropane-1-carboxylate synthase gene family in carnation. Plant Physiol. 119 (2), 755–764. 10.1104/pp.119.2.755 9952472 PMC32153

[B26] Ju C. Chang C. (2015). Mechanistic insights in ethylene perception and signal transduction. Plant Physiol. 169 (1), 85. 10.1104/pp.15.00845 26246449 PMC4577421

[B27] Kanno Y. Hanada A. Chiba Y. Ichikawa T. Nakazawa M. Matsui M. (2012). Identification of an abscisic acid transporter by functional screening using the receptor complex as a sensor. Proc. Natl. Acad. Sci. 109 (24), 9653–9658. 10.1073/pnas.1203567109 22645333 PMC3386071

[B28] Karimi M. Inze D. Depicker A. (2002). Gateway vectors for *Agrobacterium*-mediated plant transformation. Trends Plant Sci. 7 (5), 193–195. 10.1016/S1360-1385(02)02251-3 11992820

[B29] Kende H. Van Der Knaap E. Cho H.-T. (1998). Deepwater rice: a model plant to study stem elongation. Plant Physiol. 118 (4), 1105–1110. 10.1104/pp.118.4.1105 9847084 PMC1539197

[B30] Kong D. Hu H.-C. Okuma E. Lee Y. Lee H. S. Munemasa S. (2016). L-Met activates *Arabidopsis* GLR Ca^2+^ channels upstream of ROS production and regulates stomatal movement. Cell Rep. 17 (10), 2553–2561. 10.1016/j.celrep.2016.11.015 27926860

[B31] Krouk G. Lacombe B. Bielach A. Perrine-Walker F. Malinska K. Mounier E. (2010). Nitrate-regulated auxin transport by NRT1.1 defines a mechanism for nutrient sensing in plants. Dev. Cell 18 (6), 927–937. 10.1016/j.devcel.2010.05.008 20627075

[B32] Lee Y. H. Tegeder M. (2004). Selective expression of a novel high-affinity transport system for acidic and neutral amino acids in the tapetum cells of *Arabidopsis* flowers. Plant J. 40 (1), 60–74. 10.1111/j.1365-313X.2004.02186.x 15361141

[B33] Lin Z. F. Zhong S. L. Grierson D. (2009). Recent advances in ethylene research. J. Exp. Bot. 60 (12), 3311–3336. 10.1093/jxb/erp204 19567479

[B34] Liu G. Ji Y. Bhuiyan N. H. Pilot G. Selvaraj G. Zou J. (2010). Amino acid homeostasis modulates salicylic acid–associated redox status and defense responses in *Arabidopsis*. Plant Cell 22 (11), 3845–3863. 10.1105/tpc.110.079392 21097712 PMC3015111

[B35] Livak K. J. Schmittgen T. D. (2001). Analysis of relative gene expression data using real-time quantitative PCR and the 2–∆∆CT method. Methods 25 (4), 402–408. 10.1006/meth.2001.1262 11846609

[B36] Luna E. van Hulten M. Zhang Y. Berkowitz O. López A. Pétriacq P. (2014). Plant perception of β-aminobutyric acid is mediated by an aspartyl-tRNA synthetase. Nat. Chem. Biol. 10, 450. 10.1038/nchembio.1520 24776930 PMC4028204

[B37] Lurssen K. (1981). Interference of amino-acids with the uptake of 1-aminocyclopropane-1-carboxylic acid in soybean leaf-disks. Plant Sci. Lett. 20 (4), 365–370. 10.1016/0304-4211(81)90252-2

[B38] Merchante C. Stepanova A. N. (2017). “The triple response assay and its use to characterize ethylene mutants in *Arabidopsis*,” in Ethylene signaling: methods and protocols. Eds. Binder B. M. Schaller G. E. (New York, NY: Springer New York), 163–209. 10.1007/978-1-4939-6854-1_13 28293847

[B39] Métraux J.-P. Kende H. (1983). The role of ethylene in the growth response of submerged deep water rice. Plant Physiol. 72 (2), 441–446. 10.1104/pp.72.2.441 16663022 PMC1066253

[B40] Morris D. A. Larcombe N. J. (1995). Phloem transport and conjugation of foliar-applied 1-aminocyclopropane-1-carboxylic acid in cotton (*Gossypium-Hirsutum* L). J. Plant Physiol. 146 (4), 429–436. 10.1016/S0176-1617(11)82004-3

[B41] Naik B. S. (2019). Developments in taxol production through endophytic fungal biotechnology: a review. Orient. Pharm. Exp. Med. 19 (1), 1–13. 10.1007/s13596-018-0352-8

[B42] Nakatsuka A. Murachi S. Okunishi H. Shiomi S. Nakano R. Kubo Y. (1998). Differential expression and internal feedback regulation of 1-aminocyclopropane-1-carboxylate synthase, 1-aminocyclopropane-1-carboxylate oxidase, and ethylene receptor genes in tomato fruit during development and ripening. Plant Physiol. 118 (4), 1295–1305. 10.1104/pp.118.4.1295 9847103 PMC34745

[B43] Park J. Lee Y. Martinoia E. Geisler M. (2017). Plant hormone transporters: what we know and what we would like to know. BMC Biol. 15 (1), 93. 10.1186/s12915-017-0443-x 29070024 PMC5655956

[B44] Pratelli R. Pilot G. (2014). Regulation of amino acid metabolic enzymes and transporters in plants. J. Exp. Bot. 65 (19), 5535–5556. 10.1093/jxb/eru320 25114014

[B45] Qi Z. Stephens N. R. Spalding E. P. (2006). Calcium entry mediated by GLR3.3, an *Arabidopsis* glutamate receptor with a broad agonist profile. Plant Physiol. 142 (3), 963–971. 10.1104/pp.106.088989 17012403 PMC1630757

[B46] Ramesh S. A. Tyerman S. D. Xu B. Bose J. Kaur S. Conn V. (2015). GABA signalling modulates plant growth by directly regulating the activity of plant-specific anion transporters. Nat. Commun. 6, 7879. 10.1038/ncomms8879 26219411 PMC4532832

[B47] Rudus I. Sasiak M. Kepczynski J. (2013). Regulation of ethylene biosynthesis at the level of 1-aminocyclopropane-1-carboxylate oxidase (ACO) gene. Acta Physiol. Plant. 35 (2), 295–307. 10.1007/s11738-012-1096-6

[B48] Saftner R. A. Baker J. E. (1987). Transport and compartmentation of 1-aminocyclopropane-1-carboxylic acid and its structural analog, alpha-aminoisobutyric acid, in tomato pericarp slices. Plant Physiol. 84 (2), 311–317. 10.1104/pp.84.2.311 16665436 PMC1056576

[B49] Saftner R. A. Martin M. N. (1993). Transport of 1-aminocyclopropane-1-carboxylic acid into isolated maize mesophyll vacuoles. Physiol. Plant. 87 (4), 535–543. 10.1111/j.1399-3054.1993.tb02504.x

[B50] Shi L. Wu Y. Sheen J. (2018). TOR signaling in plants: conservation and innovation. Development 145 (13), dev160887. 10.1242/dev.160887 29986898 PMC6053665

[B51] Shin K. Lee S. Song W. Y. Lee R. A. Lee I. Ha K. (2015). Genetic identification of ACC-RESISTANT2 reveals involvement of lysine histidine transporter1 in the uptake of 1-aminocyclopropane-1-carboxylic acid in *Arabidopsis thaliana*. Plant Cell Physiol. 56 (3), 572–582. 10.1093/pcp/pcu201 25520403

[B52] Smith S. M. Li C. Li J. (2017). “1 - Hormone function in plants,” in Hormone metabolism and signaling in plants. Eds. Li J. Li C. Smith S. M. (Academic Press), 1–38. 10.1016/B978-0-12-811562-6.00001-3

[B53] Svennerstam H. Ganeteg U. Bellini C. Nasholm T. (2007). Comprehensive screening of *Arabidopsis* mutants suggests the lysine histidine transporter 1 to be involved in plant uptake of amino acids. Plant Physiol. 143 (4), 1853–1860. 10.1104/pp.106.092205 17293438 PMC1851813

[B54] Tegeder M. Masclaux-Daubresse C. (2018). Source and sink mechanisms of nitrogen transport and use. New Phytol. 217 (1), 35–53. 10.1111/nph.14876 29120059

[B55] Tophof S. Martinoia E. Kaiser G. Hartung W. Amrhein N. (1989). Compartmentation and transport of 1-aminocyclopropane-1-carboxylic acid and *N*-malonyl-1-aminocyclopropane-1-carboxylic acid in barley and wheat mesophyll-cells and protoplasts. Physiol. Plant. 75 (3), 333–339. 10.1111/j.1399-3054.1989.tb04635.x

[B56] Tsang D. L. Edmond C. Harrington J. L. Nühse T. S. (2011). Cell wall integrity controls root elongation *via a* general 1-aminocyclopropane-1-carboxylic acid–dependent, ethylene-independent pathway. Plant Physiol. 156 (2), 596–604. 10.1104/pp.111.175372 21508182 PMC3177261

[B57] Tsay Y.-F. Schroeder J. I. Feldmann K. A. Crawford N. M. (1993). The herbicide sensitivity gene CHL1 of arabidopsis encodes a nitrate-inducible nitrate transporter. Cell 72 (5), 705–713. 10.1016/0092-8674(93)90399-B 8453665

[B58] Tsuchisaka A. Yu G. Jin H. Alonso J. M. Ecker J. R. Zhang X. (2009). A combinatorial interplay among the 1-aminocyclopropane-1-carboxylate isoforms regulates ethylene biosynthesis in *Arabidopsis thaliana*. Genetics 183 (3), 979–1003. 10.1534/genetics.109.107102 19752216 PMC2778992

[B59] Van de Poel B. Van Der Straeten D. (2014). 1-Aminocyclopropane-1-carboxylic acid (ACC) in plants: more than just the precursor of ethylene! Front. Plant Sci. 5, 640. 10.3389/fpls.2014.00640 25426135 PMC4227472

[B60] Vanderstraeten L. Van Der Straeten D. (2017). Accumulation and transport of 1-aminocyclopropane-1-carboxylic acid (ACC) in plants: current status, considerations for future research and agronomic applications. Front. Plant Sci. 8, 38. 10.3389/fpls.2017.00038 28174583 PMC5258695

[B61] Vriezen W. H. Zhou Z. Van Der Straeten D. (2003). Regulation of submergence-induced enhanced shoot elongation in *Oryza sativa* L. Ann. Bot. 91 (2), 263–270. 10.1093/aob/mcf121 12509346 PMC4244991

[B62] Woltering E. J. (1990). Interorgan translocation of 1-aminocyclopropane-1-carboxylic acid and ethylene coordinates senescence in emasculated *Cymbidium* flowers. Plant physiol. 92 (3), 837–845. 10.1104/pp.92.3.837 16667357 PMC1062377

[B63] Xu S.-L. Rahman A. Baskin T. I. Kieber J. J. (2008). Two leucine-rich repeat receptor kinases mediate signaling, linking cell wall biosynthesis and ACC synthase in *Arabidopsis*. Plant Cell 20 (11), 3065–3079. 10.1105/tpc.108.063354 19017745 PMC2613664

[B64] Yang S. F. Hoffman N. E. (1984). Ethylene biosynthesis and its regulation in higher-plants. Annu. Rev. Plant Physiol. Plant Mol. Biol. 35, 155–189. 10.1146/annurev.pp.35.060184.001103

[B65] Yoon G. M. Kieber J. J. (2013). 1-Aminocyclopropane-1-carboxylic acid as a signalling molecule in plants. AoB Plants 5, plt017. 10.1093/aobpla/plt017

