# Supplementary material for: Identification of Lysine Histidine Transporter 2 as an 1-Aminocyclopropane Carboxylic Acid Transporter in Arabidopsis thaliana by Transgenic Complementation Approach
Source: Front Plant Sci. 2019 Sep 11;10:1092. doi: 10.3389/fpls.2019.01092 (PMC6749071; doi:10.3389/fpls.2019.01092)
Supplement: Supplementary file 1 [file Presentation_1.pptx]

## Slide 1
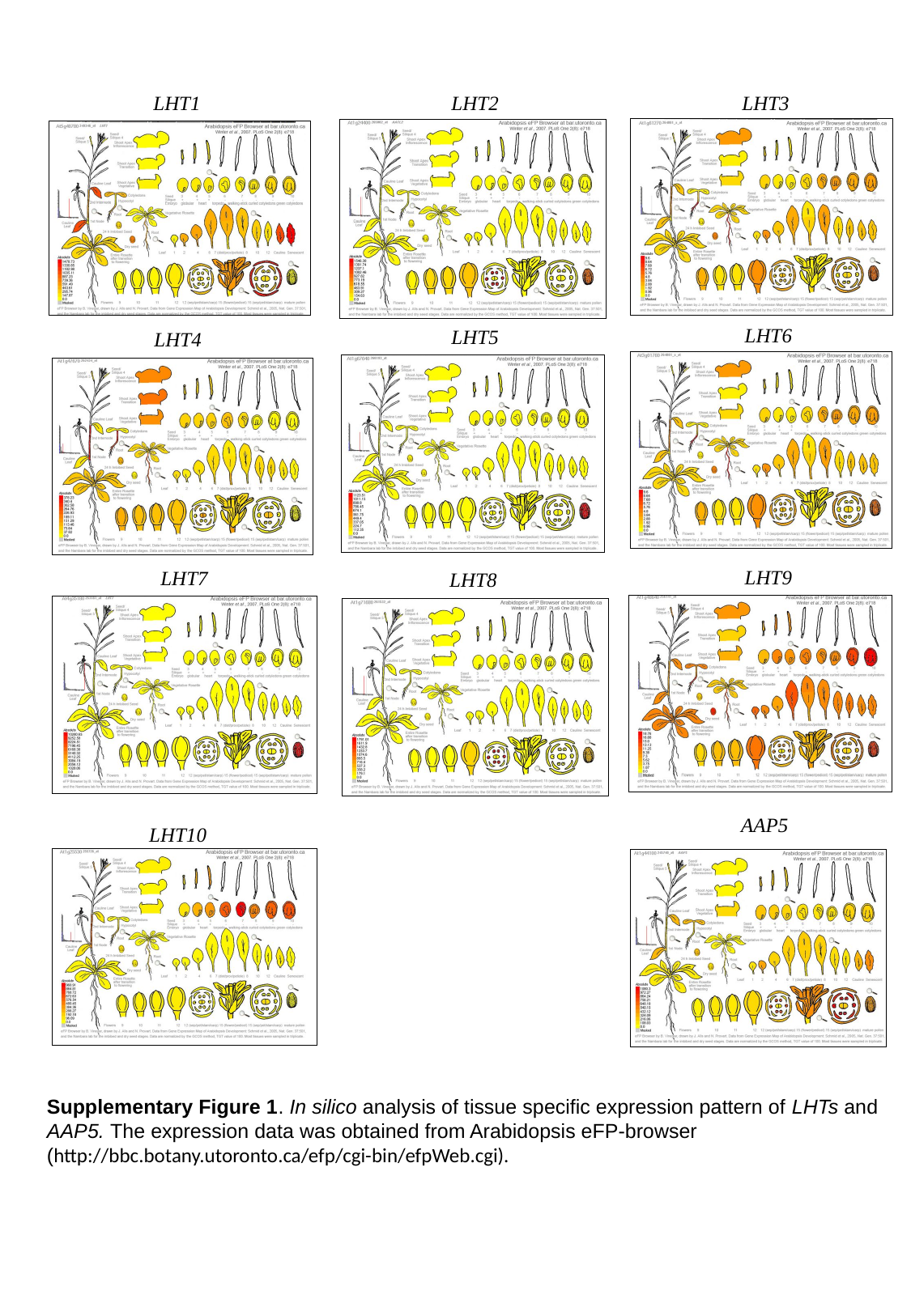

LHT1
LHT3
LHT2
LHT6
LHT5
LHT4
LHT9
LHT7
LHT8
LHT10
AAP5
Supplementary Figure 1. In silico analysis of tissue specific expression pattern of LHTs and AAP5. The expression data was obtained from Arabidopsis eFP-browser (http://bbc.botany.utoronto.ca/efp/cgi-bin/efpWeb.cgi).

## Slide 2
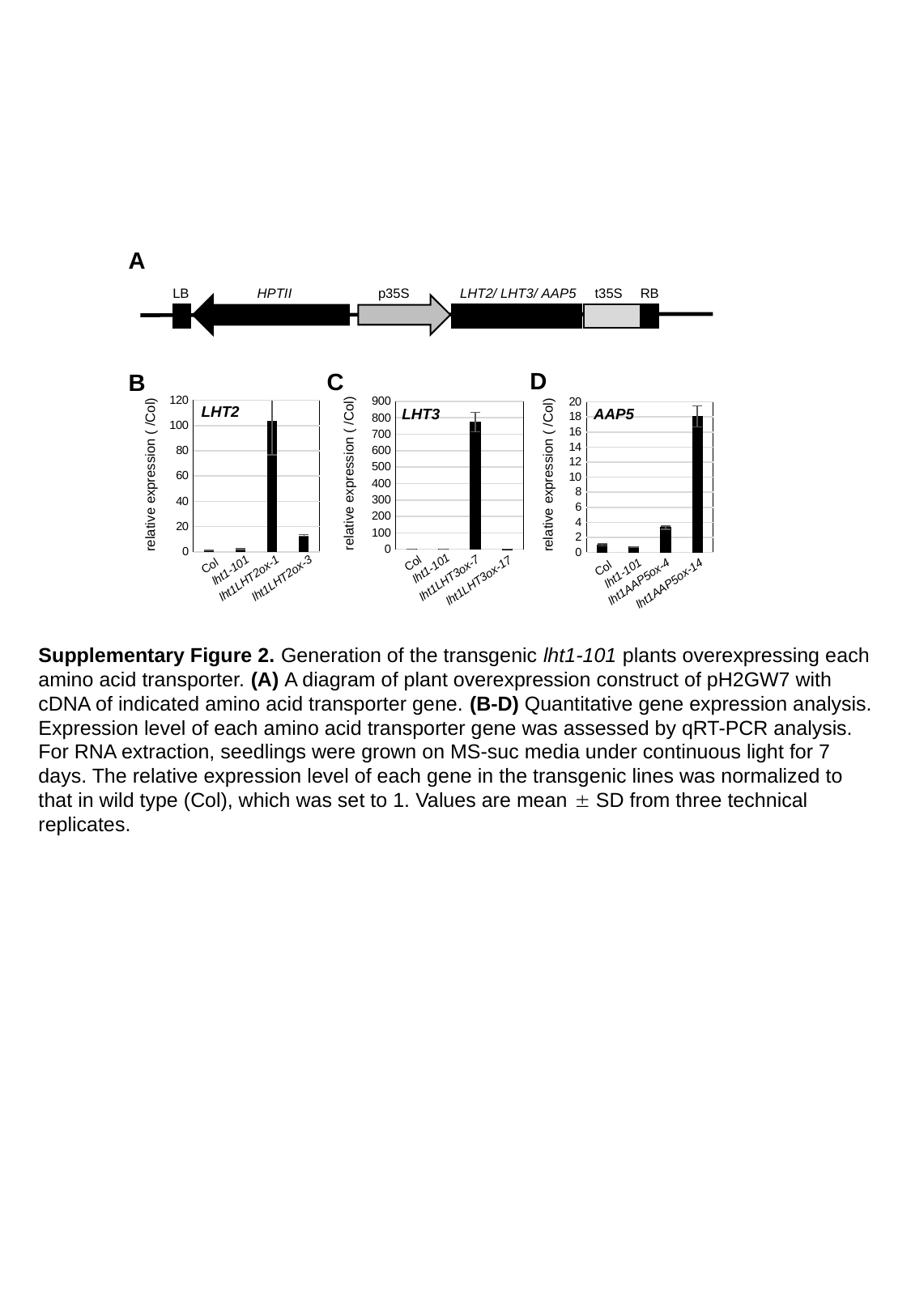

A
LB
HPTII
p35S
LHT2/ LHT3/ AAP5
t35S
RB
D
C
B
### Chart
| Category | |
|---|---|
| Col | 1.006520796444428 |
| are2 | 0.7141449916717795 |
| AAP5 ox 4 | 3.300291455171054 |
| AAP5 ox 14 | 18.079802160683048 |
### Chart
| Category | |
|---|---|
| Col | 1.0785339170168828 |
| are2 | 0.6172346555297443 |
| are2LHT3ox-7 | 773.3687945754256 |
| are2LHT3ox-17 | 0.5636859381781472 |
### Chart
| Category | |
|---|---|
| Col | 1.017859471704975 |
| lht1are2 | 2.356933601542543 |
| are2LHT2ox-1 | 103.65108963189033 |
| are2LHT2ox-3 | 12.999352464509577 |LHT2
LHT3
AAP5
relative expression ( /Col)
relative expression ( /Col)
relative expression ( /Col)
Col
Col
lht1-101
Col
lht1-101
lht1-101
lht1LHT3ox-7
lht1LHT2ox-1
lht1LHT2ox-3
lht1LHT3ox-17
lht1AAP5ox-4
lht1AAP5ox-14
Supplementary Figure 2. Generation of the transgenic lht1-101 plants overexpressing each amino acid transporter. (A) A diagram of plant overexpression construct of pH2GW7 with cDNA of indicated amino acid transporter gene. (B-D) Quantitative gene expression analysis. Expression level of each amino acid transporter gene was assessed by qRT-PCR analysis. For RNA extraction, seedlings were grown on MS-suc media under continuous light for 7 days. The relative expression level of each gene in the transgenic lines was normalized to that in wild type (Col), which was set to 1. Values are mean ± SD from three technical replicates.

## Slide 3
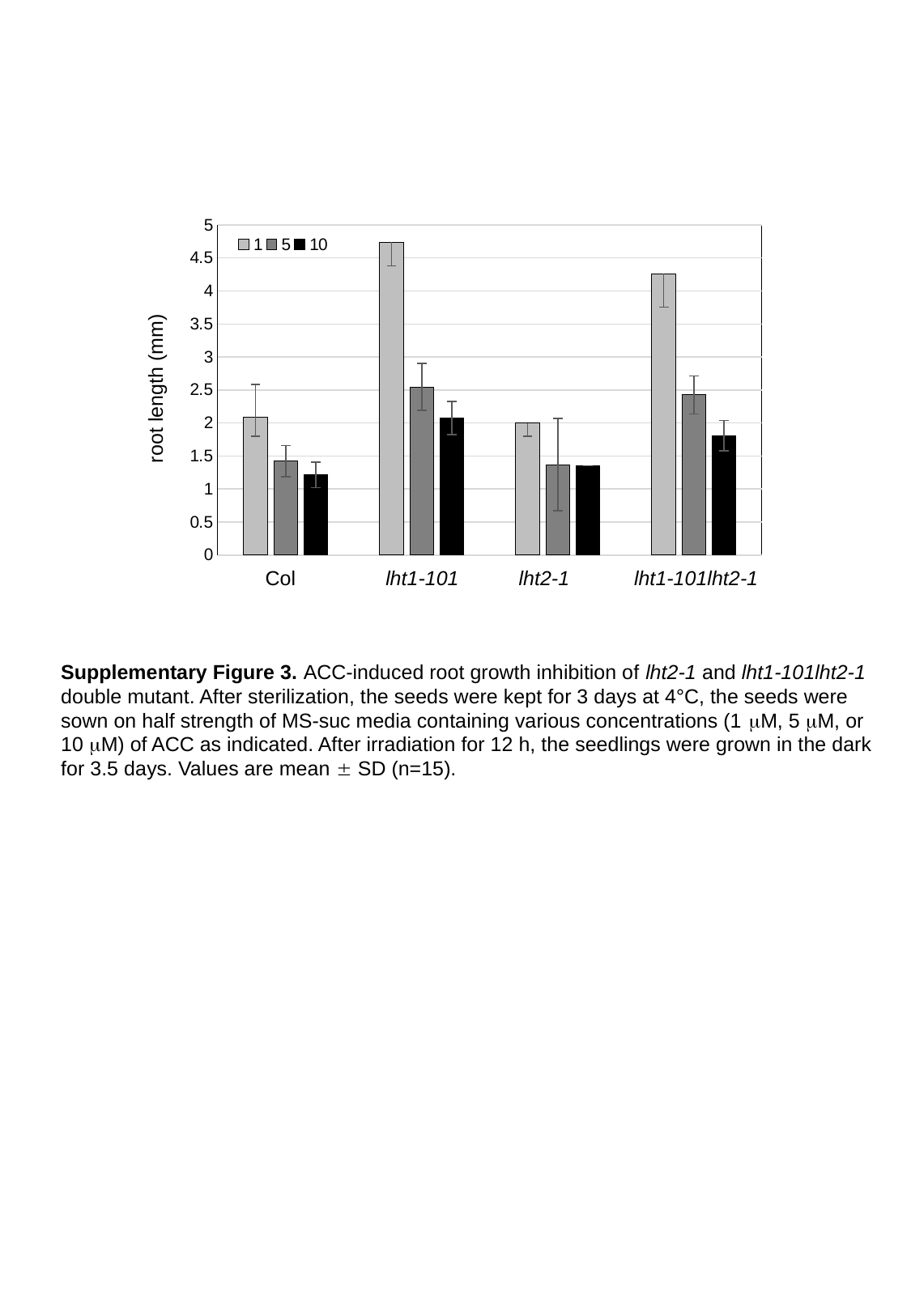

### Chart
| Category | 1 | 5 | 10 |
|---|---|---|---|
| col | 2.0825 | 1.424 | 1.212 |
| are2 | 4.7379999999999995 | 2.5473333333333334 | 2.0780000000000003 |
| lht2-1 | 2.0 | 1.37 | 1.35 |
| are2lht2-1 | 4.262666666666667 | 2.425625 | 1.809333333333333 |root length (mm)
lht1-101
Col
lht2-1
lht1-101lht2-1
Supplementary Figure 3. ACC-induced root growth inhibition of lht2-1 and lht1-101lht2-1 double mutant. After sterilization, the seeds were kept for 3 days at 4°C, the seeds were sown on half strength of MS-suc media containing various concentrations (1 μM, 5 μM, or 10 μM) of ACC as indicated. After irradiation for 12 h, the seedlings were grown in the dark for 3.5 days. Values are mean ± SD (n=15).

## Slide 4
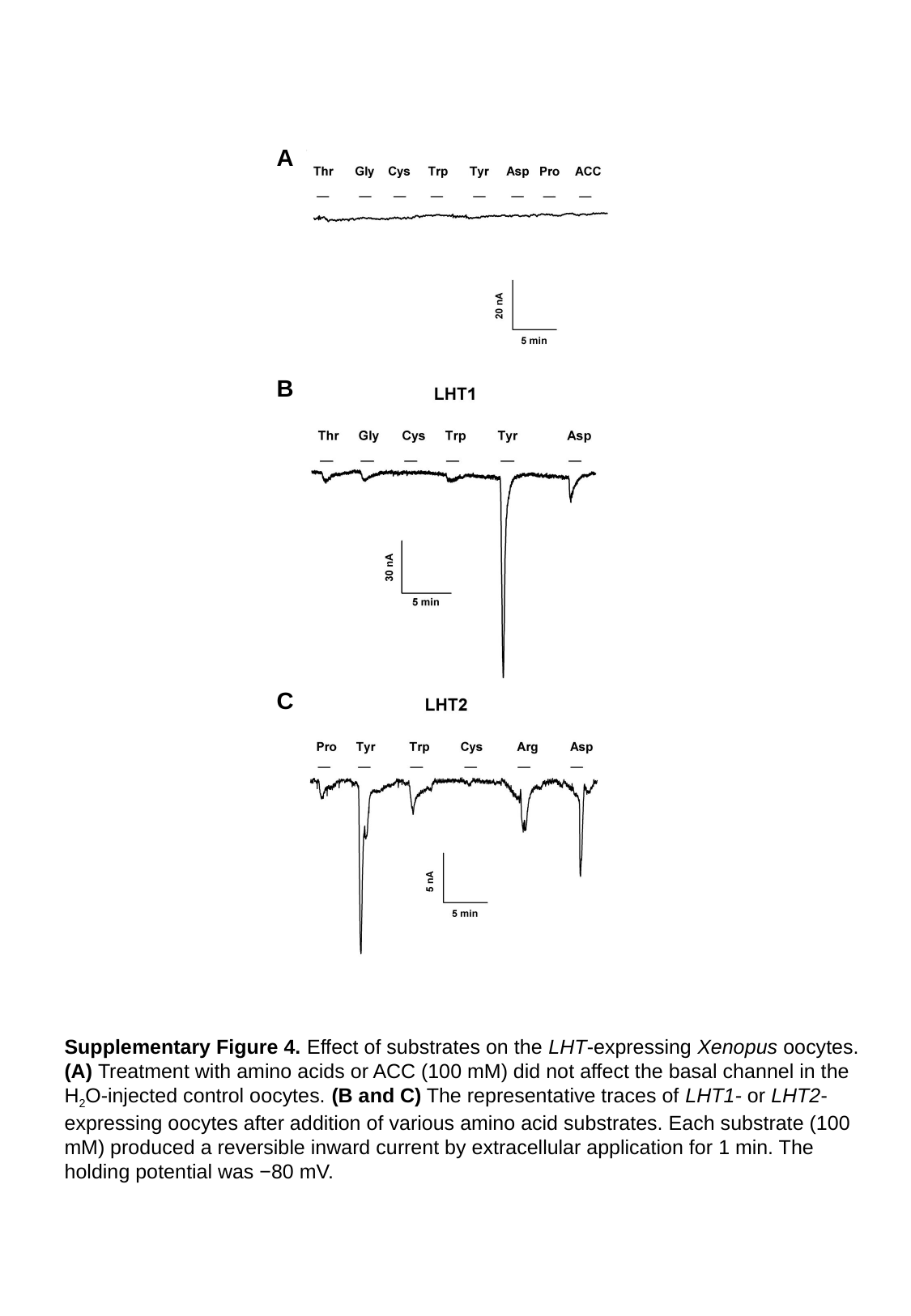

A
B
C
Supplementary Figure 4. Effect of substrates on the LHT-expressing Xenopus oocytes. (A) Treatment with amino acids or ACC (100 mM) did not affect the basal channel in the H2O-injected control oocytes. (B and C) The representative traces of LHT1- or LHT2-expressing oocytes after addition of various amino acid substrates. Each substrate (100 mM) produced a reversible inward current by extracellular application for 1 min. The holding potential was −80 mV.

## Slide 5
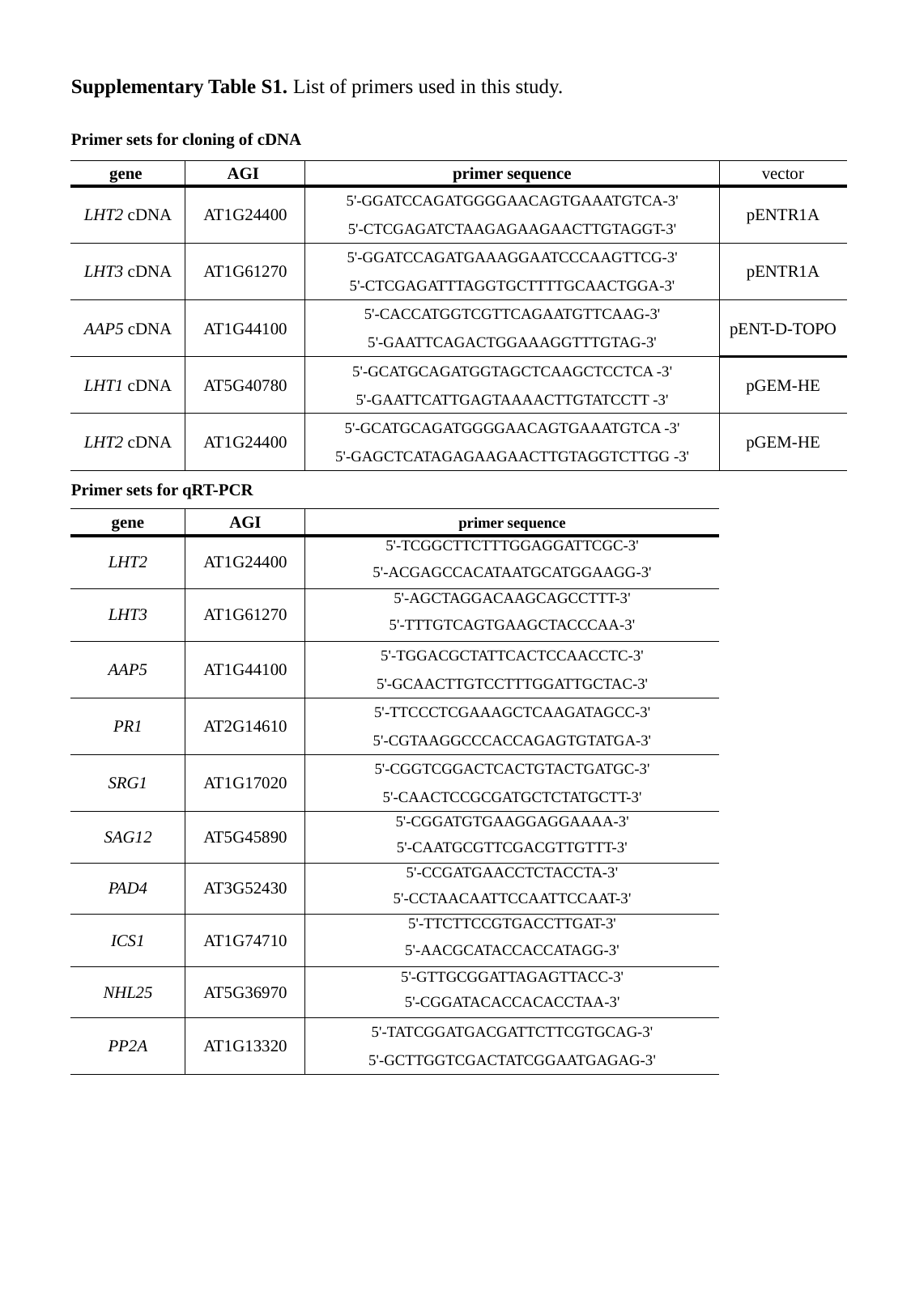

| Supplementary Table S1. List of primers used in this study. | | | | | |
| --- | --- | --- | --- | --- | --- |
| | | | | | |
| Primer sets for cloning of cDNA | | | | | |
| gene | AGI | primer sequence | | | vector |
| LHT2 cDNA | AT1G24400 | 5'-GGATCCAGATGGGGAACAGTGAAATGTCA-3' | | | pENTR1A |
| | | 5'-CTCGAGATCTAAGAGAAGAACTTGTAGGT-3' | | | |
| LHT3 cDNA | AT1G61270 | 5'-GGATCCAGATGAAAGGAATCCCAAGTTCG-3' | | | pENTR1A |
| | | 5'-CTCGAGATTTAGGTGCTTTTGCAACTGGA-3' | | | |
| AAP5 cDNA | AT1G44100 | 5'-CACCATGGTCGTTCAGAATGTTCAAG-3' | | | pENT-D-TOPO |
| | | 5'-GAATTCAGACTGGAAAGGTTTGTAG-3' | | | |
| LHT1 cDNA | AT5G40780 | 5'-GCATGCAGATGGTAGCTCAAGCTCCTCA -3' | | | pGEM-HE |
| | | 5'-GAATTCATTGAGTAAAACTTGTATCCTT -3' | | | |
| LHT2 cDNA | AT1G24400 | 5'-GCATGCAGATGGGGAACAGTGAAATGTCA -3' | | | pGEM-HE |
| | | 5'-GAGCTCATAGAGAAGAACTTGTAGGTCTTGG -3' | | | |
| Primer sets for qRT-PCR | | | | | |
| gene | AGI | primer sequence | | | |
| LHT2 | AT1G24400 | 5'-TCGGCTTCTTTGGAGGATTCGC-3' | | | |
| | | 5'-ACGAGCCACATAATGCATGGAAGG-3' | | | |
| LHT3 | AT1G61270 | 5'-AGCTAGGACAAGCAGCCTTT-3' | | | |
| | | 5'-TTTGTCAGTGAAGCTACCCAA-3' | | | |
| AAP5 | AT1G44100 | 5'-TGGACGCTATTCACTCCAACCTC-3' | | | |
| | | 5'-GCAACTTGTCCTTTGGATTGCTAC-3' | | | |
| PR1 | AT2G14610 | 5'-TTCCCTCGAAAGCTCAAGATAGCC-3' | | | |
| | | 5'-CGTAAGGCCCACCAGAGTGTATGA-3' | | | |
| SRG1 | AT1G17020 | 5'-CGGTCGGACTCACTGTACTGATGC-3' | | | |
| | | 5'-CAACTCCGCGATGCTCTATGCTT-3' | | | |
| SAG12 | AT5G45890 | 5'-CGGATGTGAAGGAGGAAAA-3' | | | |
| | | 5'-CAATGCGTTCGACGTTGTTT-3' | | | |
| PAD4 | AT3G52430 | 5'-CCGATGAACCTCTACCTA-3' | | | |
| | | 5'-CCTAACAATTCCAATTCCAAT-3' | | | |
| ICS1 | AT1G74710 | 5'-TTCTTCCGTGACCTTGAT-3' | | | |
| | | 5'-AACGCATACCACCATAGG-3' | | | |
| NHL25 | AT5G36970 | 5'-GTTGCGGATTAGAGTTACC-3' | | | |
| | | 5'-CGGATACACCACACCTAA-3' | | | |
| PP2A | AT1G13320 | 5'-TATCGGATGACGATTCTTCGTGCAG-3' | | | |
| | | 5'-GCTTGGTCGACTATCGGAATGAGAG-3' | | | |

## Slide 6
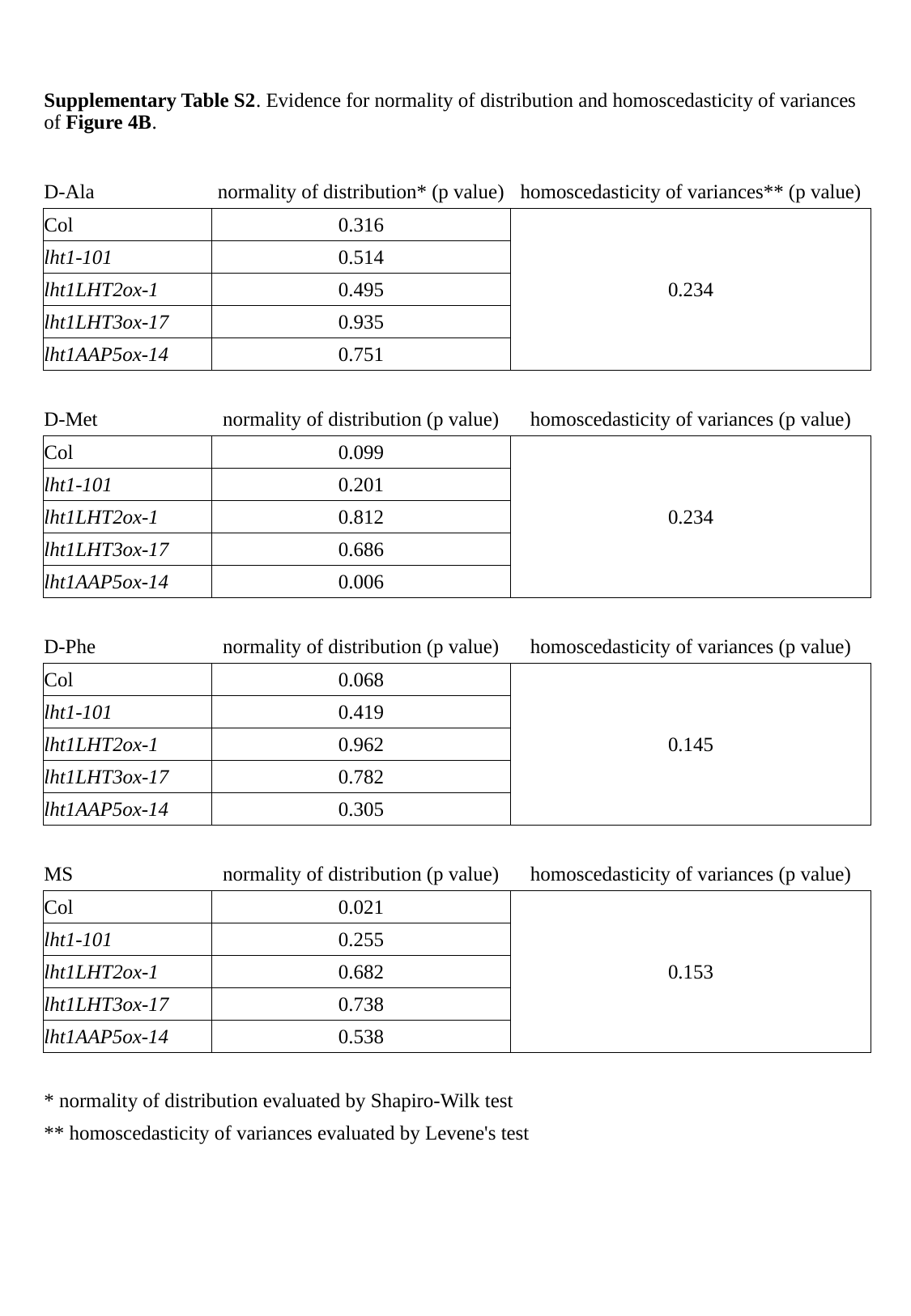

| Supplementary Table S2. Evidence for normality of distribution and homoscedasticity of variances of Figure 4B. | | |
| --- | --- | --- |
| | | |
| D-Ala | normality of distribution\* (p value) | homoscedasticity of variances\*\* (p value) |
| Col | 0.316 | 0.234 |
| lht1-101 | 0.514 | |
| lht1LHT2ox-1 | 0.495 | |
| lht1LHT3ox-17 | 0.935 | |
| lht1AAP5ox-14 | 0.751 | |
| | | |
| D-Met | normality of distribution (p value) | homoscedasticity of variances (p value) |
| Col | 0.099 | 0.234 |
| lht1-101 | 0.201 | |
| lht1LHT2ox-1 | 0.812 | |
| lht1LHT3ox-17 | 0.686 | |
| lht1AAP5ox-14 | 0.006 | |
| | | |
| D-Phe | normality of distribution (p value) | homoscedasticity of variances (p value) |
| Col | 0.068 | 0.145 |
| lht1-101 | 0.419 | |
| lht1LHT2ox-1 | 0.962 | |
| lht1LHT3ox-17 | 0.782 | |
| lht1AAP5ox-14 | 0.305 | |
| | | |
| MS | normality of distribution (p value) | homoscedasticity of variances (p value) |
| Col | 0.021 | 0.153 |
| lht1-101 | 0.255 | |
| lht1LHT2ox-1 | 0.682 | |
| lht1LHT3ox-17 | 0.738 | |
| lht1AAP5ox-14 | 0.538 | |
| | | |
| \* normality of distribution evaluated by Shapiro-Wilk test | | |
| \*\* homoscedasticity of variances evaluated by Levene's test | | |
